# Serotype, antibiotic susceptibility and whole-genome characterization of *Streptococcus pneumoniae* in all age groups living in Southwest China during 2018–2022

**DOI:** 10.3389/fmicb.2024.1342839

**Published:** 2024-02-01

**Authors:** Chenglin Miao, Ziyi Yan, Chunmei Chen, Linghan Kuang, Keping Ao, Yingying Li, Jialu Li, Xiaocui Huang, Xinghua Zhu, Yijia Zhao, Yali Cui, Yongmei Jiang, Yi Xie

**Affiliations:** ^1^Department of Laboratory Medicine, West China Second University Hospital, Sichuan University, Chengdu, Sichuan, China; ^2^Department of Laboratory Medicine, Meishan Women and Children’s Hospital, Alliance Hospital of West China Second University Hospital, Sichuan University, Meishan, Sichuan, China; ^3^Department of Laboratory Medicine, West China Second University Hospital (Tianfu), Sichuan University/Sichuan Provincial Children’s Hospital, Meishan, Sichuan, China; ^4^Department of Laboratory Medicine, West China Hospital, Sichuan University, Chengdu, Sichuan, China; ^5^Department of Laboratory Medicine, Tibet Autonomous Region Women's and Children's Hospital, Lhasa, China; ^6^Department of Obstetrics, Key Laboratory of Birth Defects and Related Disease of Women and Children of MOE, West China Second Hospital, Sichuan University, Chengdu, Sichuan, China; ^7^Department of Laboratory Medicine, Chengdu Jinjiang District Maternal and Child Healthcare Hospital, Chengdu, Sichuan, China; ^8^Department of Laboratory Medicine, The First People’s Hospital of Longquanyi District, Chengdu, Sichuan, China; ^9^Key Laboratory of Birth Defects and Related Diseases of Women and Children (Sichuan University), Ministry of Education, Chengdu, Sichuan, China

**Keywords:** *Streptococcus pneumoniae*, serotype, molecular characterization, antibiotic resistance, whole-genome sequencing, China

## Abstract

**Background:**

*Streptococcus pneumoniae* is a common pathogen that colonizes the human upper respiratory tract, causing high morbidity and mortality worldwide. This study aimed to investigate the prevalence status of *S. pneumoniae* isolated from patients of all ages in Southwest China, including serotype, antibiotic susceptibility and other molecular characteristics, to provide a basis for clinical antibiotic usage and vaccine development.

**Methods:**

This study was conducted from January 2018 to March 2022 at West China Hospital, West China Second University Hospital, First People’s Hospital of Longquanyi District (West China Longquan Hospital), Meishan Women and Children’s Hospital (Alliance Hospital of West China Second University Hospital) and Chengdu Jinjiang Hospital for Women and Children Health. Demographic and clinical characteristics of 263 pneumococcal disease (PD) all-age patients were collected and analyzed. The serotypes, sequence types (STs), and antibiotic resistance of the strains were determined by next-generation sequencing, sequence analysis and the microdilution broth method.

**Results:**

The most common pneumococcal serotypes were 19F (17.87%), 19A (11.41%), 3 (8.75%), 23F (6.46%) and 6A (5.70%). Coverage rates for PCV10, PCV13, PCV15, PCV20 and PCV24 were 36.12, 61.98, 61.98, 63.12 and 64.26%, respectively. Prevalent STs were ST271 (12.55%), ST320 (11.79%), ST90 (4.18%), ST876 (4.18%) and ST11972 (3.42%). Penicillin-resistant *S. pneumoniae* (PRSP) accounted for 82.35 and 1.22% of meningitis and nonmeningitis PD cases, respectively. Resistance genes *msrD* (32.7%), *mefA* (32.7%), *ermB* (95.8%), *tetM* (97.3%) and *catTC* (7.6%) were found among 263 isolates. Most isolates showed high resistance to erythromycin (96.96%) and tetracycline (79.85%), with more than half being resistant to SXT (58.94%). A few isolates were resistant to AMX (9.89%), CTX (11.03%), MEN (9.13%), OFX (1.14%), LVX (1.14%) and MXF (0.38%). All isolates were susceptible to vancomycin and linezolid.

**Conclusion:**

Our study provides reliable information, including the prevalence, molecular characterization and antimicrobial resistance of *S. pneumoniae* isolates causing pneumococcal diseases in Southwest China. The findings contribute to informed and clinical policy decisions for prevention and treatment.

## 1 Introduction

*Streptococcus pneumoniae* is a common gram-positive, opportunistic pathogen in humans that colonizes the nasopharynx. It can cause not only noninvasive pneumococcal diseases (NIPDs), such as pneumonia, otitis media, conjunctivitis, nasosinusitis and bronchitis, but also severe invasive pneumococcal diseases (IPDs), including pleurisy, meningitis and septicemia (Zeng et al., 2023), especially in children and elderly individuals. According to the World Health Organization (WHO), *S. pneumoniae* is the most common pathogen causing pneumonia. More than approximately 800,000 children die due to pneumococcal diseases annually, and the vast majority are in developing and underdeveloped countries (O'Brien et al., 2009; Johnson et al., 2010). Furthermore, IPDs are age influenced, with incidence increasing with age and mortality higher in those over 65 years of age (Marrie et al., 2018).

There are currently more than 100 known *S. pneumoniae* serotypes circulating worldwide (Ganaie et al., 2020). However, only some of these serotypes cause pneumococcal disease (Namkoong et al., 2016). Currently, several countries have already incorporated pneumococcal conjugate vaccines (PCVs) and polysaccharide-based vaccines (PPVs) into national immunization programs, and use of vaccines targeting specific serotypes has significantly reduced IPDs caused by the vaccine serotypes (VTs) (Richter et al., 2014; Chan et al., 2019). Vaccines such as PCV7 (covering serotypes 4, 6B, 9 V, 14, 18C, 19F and 23F), PCV13 (additionally covering 1, 3, 5, 6A, 7F and 19A), and PPV23 (covering serotypes 1–5, 6B, 7F, 8, 9 N, 9 V, 10A, 11A, 12F, 14, 15B, 17F, 18C, 19A, 19F, 20, 22F, 23F, and 33F) are currently available in China. However, none of the above vaccines is a part of China National Vaccination Programs, and the cost is high. These reasons result in low coverage rates (Men et al., 2020; Wang et al., 2021). In general, use of vaccines has led to changes in the distribution of *S. pneumoniae* serotypes. Although these vaccines have provided protection against VTs, they have also contributed to a rise in noncovered serotypes (NVTs; Lo et al., 2022). The increase in NVTs is concerning, especially as these serotypes display resistance to antibiotics commonly used in PD treatment (Zhao et al., 2020).

Antibiotic resistance varies by region, and a better understanding of resistance will ultimately help in clinical anti-infective treatment and preventing the spread of antibiotic-resistant strains (Li et al., 2023). Meanwhile, the distribution of *S. pneumoniae* serotypes in a specific region can provide valuable evidence for local health authorities to introduce appropriate vaccines. Analysis of whole-genome sequencing technology based on next-generation sequencing (NGS) can obtain information about molecular serotype, sequence type (ST), and antibiotic resistance, providing an important basis for pathogenic surveillance.

Furthermore, research shows that the coronavirus disease 2019 (COVID-19) pandemic and subsequent lockdowns to interrupt the spread of the virus had an impact on the pathogenicity of *S. pneumoniae* (Amin-Chowdhury et al., 2021). Despite PD cases have declined significantly during the COVID-19 pandemic. However, the mortality rate of IPD/COVID-19 co-infection is very high (Mitsi et al., 2022). Therefore, molecular characterization of prevalent *S. pneumoniae* strains during the COVID-19 outbreak will provide important support and reference for investigating the current status of PDs and potential changes in subsequent isolates.

The aim of this study based on whole-genome NGS technology was to investigate the prevalence and molecular characteristics of clinical *S. pneumoniae* isolates obtained from patients of all ages in Southwest China from 2018 to 2022 by analyzing the distribution of serotypes, STs, antimicrobial susceptibility and their respective relationships.

## 2 Materials and methods

### 2.1 Patients enrolled

This study was conducted from January 2018 to March 2022 at West China Hospital, West China Second University Hospital, First People’s Hospital of Longquanyi District (West China Longquan Hospital), Meishan Women and Children’s Hospital (Alliance Hospital of West China Second University Hospital) and Chengdu Jinjiang Hospital for Women and Children Health, including one of China’s largest general hospitals, one of China’s largest specialty hospitals for children and women, and typical secondary and tertiary general or specialty hospitals. The hospitals’ clinical laboratories have been accredited by the College of American Pathologists (CAP) or the China National Accreditation Service for Conformity Assessment (CNAS) under the ISO15189 accreditation standard or are under the supervision of the abovementioned external quality assessment laboratory.

The enrolled subjects were patients from Southwest China presenting with an *S. pneumoniae* infection who were admitted to these hospitals. The participant eligibility criteria included the following: (1) clinical specimens from which *S. pneumoniae* was isolated and positively cultured (including blood, cerebrospinal fluid, drainage fluid, alveolar lavage fluid, sputum, and secretions); and (2) respiratory, neural, circulatory or local infectious manifestations (including fever, headache, cough, and sputum).

### 2.2 Isolation and identification of strains

Strains of *S. pneumoniae* were collected, isolated and identified in line with requirements for clinical procedures as previously reported (Yan et al., 2019). In brief, specimens were collected by specialized sample collection personnel or physicians, following the Standard Operating Procedure (SOP) of ISO/TS 20658:2017-Medical Laboratories-Requirements^1^ for collection, transport, receipt, and handling of samples (blood, sputum, cerebrospinal fluid, chest drainage and secretions). The alveolar lavage fluid was collected by flexible bronchoscopy via the protocol of Chinese expert consensus on pathogen detection in bronchoalveolar lavage for pulmonary infectious diseases (2017 edition) (Qu et al., 2017). Blood, cerebrospinal fluid and chest drainage were cultured in vials using the BD BACTECTM FX system and then subcultured onto Columbia Agar +5% sheep blood plates (Autobio, Zhengzhou, China). Other samples were isolated on Columbia agar +5% sheep blood plates (Autobio, Zhengzhou, China) incubated at 35°C for 24–48 h in a 5% carbon dioxide (CO_2_) environment. All isolates were identified by matrix-assisted laser desorption ionization time-of-flight mass spectrometry (MALDI-TOF MS; Vitek MS system; BioMerieux, Rhône, France), and the results were confirmed by PubMLST whole-genome signature sequence alignment.^2^ The pneumococcal isolates were stored in 25% sterile glycerol broth at −70°C for subsequent analysis.

### 2.3 Genome sequencing, assembly and annotation

Genomic DNA was extracted by using QIAamp DNA Minikit (Qiagen, Hilden, Germany), and sequencing libraries were generated using NEBNext^®^ Ultra^™^ DNA Library Prep Kit for Illumina (New England Biolabs, NEB, USA) following the manufacturer’s recommendations. The whole genomes of *S. pneumoniae* were sequenced using the Illumina NovaSeq PE150 platform (Illumina, San Diego, CA, USA) with approximately 200× coverage at Beijing Novogene Bioinformatics Technology Co., Ltd.

Genome data were assembled and annotated as previously reported (Cui et al., 2022) with SPAdes software (v3.14.1) (Prjibelski et al., 2020) and Prokka software (v1.14.5) (Seemann, 2014). All of the assembled genomes were submitted to GenBank and approved (PRJNA914101, PRJNA915833 and PRJNA915821).^3^

### 2.4 Molecular serotyping and multilocus sequence typing

MLSTs were identified by pneumococcal capsule typing (PneumoCaT v1.2.1) based on genome data (*.fastq files) as previously reported (Kapatai et al., 2016). STs were identified through the multilocus sequence typing (MLST) database^4^ and mlst software (v2.19.0) (Seemann T, mlst, GitHub^5^) (Jolley and Maiden, 2010), and all novel alleles and profiles were submitted to the pneumococcal MLST database to assign new numbers. Minimum spanning tree-like structures were illustrated by PHYLOVIZ software (version 2.1)^6^ via goeBURST Full MST (goeBURST distance) at level 1 (SLVs) and level 6 (Feil et al., 2004; Francisco et al., 2012).

### 2.5 Phylogenetic analysis

The annotated pneumococcal genome data were analyzed using Roary (v3.13.0)^7^ to create a multiFASTA alignment of core genes (>99%) (Page et al., 2015), and Snp-sites (v2.3.3)^8^ were used to delete duplicate sites. Maximum-likelihood trees were constructed from the alignment produced by RAxML (v8.2.10)^9^ using the GTRGAMMA method (Stamatakis, 2014), and the RAxML trees were visualized and annotated in iTOL (v6).^10^

### 2.6 Antibiotic susceptibility tests and resistance gene screening

Antibiotic susceptibility tests (ASTs) were performed based on the broth turbidimetry method using AST dishes (TDR STR-AST, Mindray, China). The antimicrobial agents used included penicillin (PEN), amoxicillin (AMX), cefotaxime (CTX), meropenem (MEM), vancomycin (VAN), erythromycin (ERY), ofloxacin (OFX), levofloxacin (LVX), moxifloxacin (MXF), tetracycline (TET) and trimethoprim-sulfamethoxazole (SXT). Quality control analysis was performed using *S. pneumoniae* ATCC49619. The operational processes and interpretation of the results were performed according to the manufacturer’s instructions and the Clinical and Laboratory Standards Institute (CLSI) 2021 standard (Humphries et al., 2021).

Antimicrobial resistance genes were screened with ABRicate software (v1.0.1; Seemann T, Abricate, GitHub)^11^ via NCBI AMRFinderPlus (Feldgarden et al., 2019).

### 2.7 Statistical analysis

Statistical Package for Social Science (SPSS) software for Windows was used to assess the statistical significance of the data (version 22.0; Chicago, IL, USA), as previously reported (Yan et al., 2019). In brief, the chi-square test, Fisher’s exact test and T-test were used. Based on the chi-square test, the Bonferroni method was employed to determine whether differences among multiple groups were statistically significant, and *p* values <0.05 were considered statistically significant.

### 2.8 Ethics statement

The clinical experimental plan was approved by the Clinical Trial Ethics Committee of West China Second University Hospital, Sichuan University (No. 2020041). The work was carried out in accordance with the Declaration of Helsinki.

## 3 Results

### 3.1 Demographic and clinical characteristics

A total of 263 patients were enrolled from January 2018 to March 2022, including 93 patients under 3 years old (35.36%) and 101 patients above 50 years old (38.40%), with ages ranging from 0.08 to 95 years and a median age (P25-P75) of 8.00 (2.34–63.00) years. These patients were diagnosed with meningitis, bacteremia, pleurisy, otitis media, pneumonia, bronchitis and upper respiratory tract infection, including 88 IPD patients and 175 NIPD patients. The detailed data are shown in Table 1.

**Table 1:** Demographic and clinical characteristics of 263 patients.

Characteristics	No. of patients	(%)
*Sex*		
Male	166	63.12
Female	97	36.88
*Age (years)*		
≤3	93	35.36
3–18	48	18.25
18–50	21	7.98
50–95	101	38.40
*Median age (P25-P75)*	*8.00 (2.34–63.00)*	
*Isolation time of year*		
Quarter 1	60	22.81
Quarter 2	34	12.93
Quarter 3	49	18.63
Quarter 4	120	45.63
*Sample type*		
Blood	48	18.25
Cerebrospinal fluid	17	6.47
Chest drainage	12	4.56
Alveolar lavage fluid	11	4.18
Sputum	155	58.94
Nasopharyngeal swab	5	1.90
Tracheal secretions	9	3.42
Ear canal secretions	3	1.14
Others	3	1.14
*Diagnosis*		
Invasive pneumococcal disease (IPD)	88	33.46
Noninvasive pneumococcal disease (NIPD)	175	66.54
Total	263	100.00

### 3.2 Molecular serotyping and vaccine coverage rates

**Figure 1:**
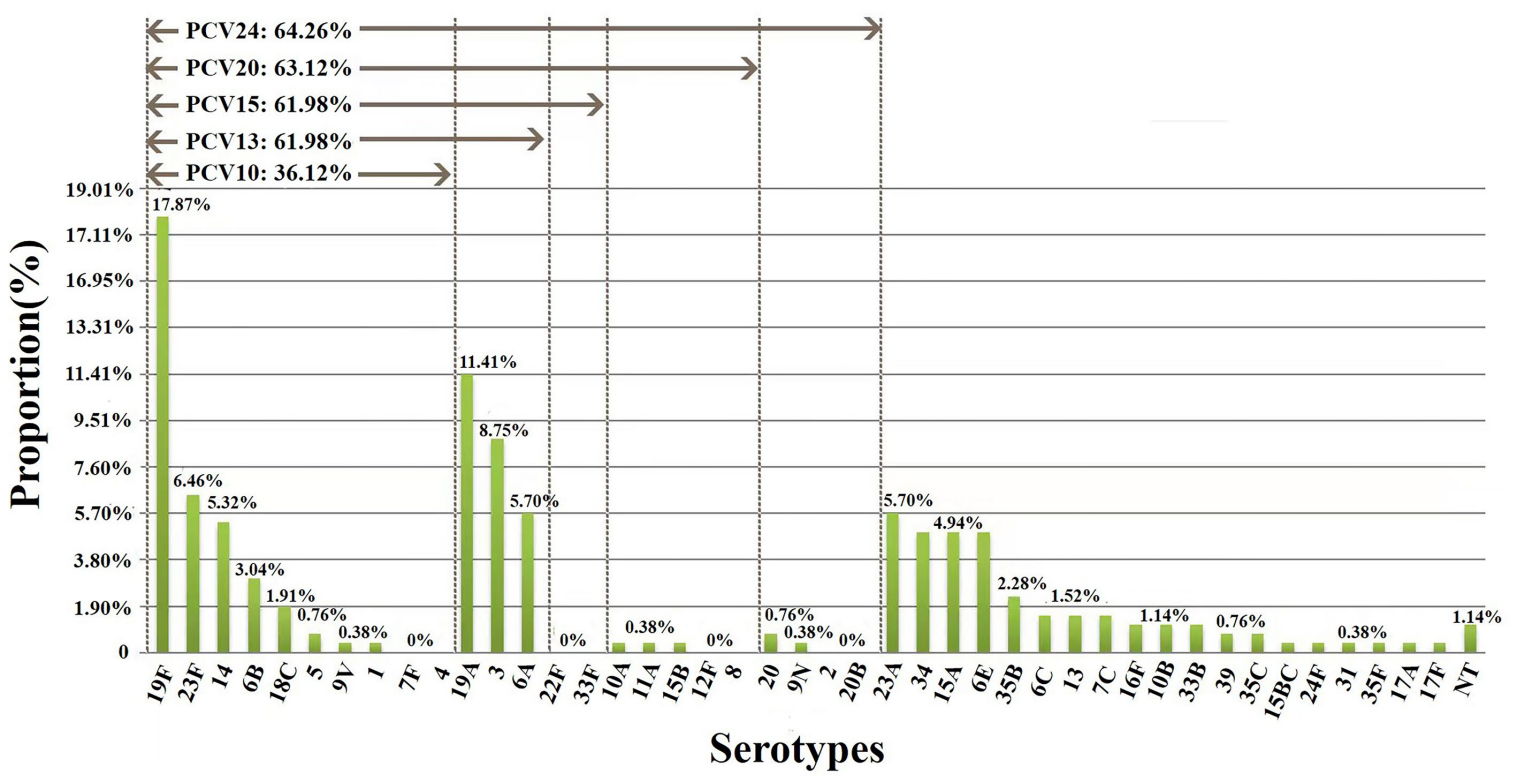
Serotype distribution and coverage of PCVs among 263 *S. pneumoniae* isolates. The prevalent serotypes were 19F (*n* = 47, 17.87%), 19A (*n* = 30, 11.41%), 3 (*n* = 23, 8.75%) and 23F (*n* = 17, 6.46%). NT, nontypable.

Among 263 isolates, 35 serotypes were successfully identified. The prevalent serotypes were 19F (*n* = 47, 17.87%), 19A (*n* = 30, 11.41%), 3 (*n* = 23, 8.75%), 23F (*n* = 17, 6.46%), 6A (*n* = 15, 5.70%), 23A (*n* = 15, 5.70%), 14 (*n* = 14, 5.32%), 34 (*n* = 13, 4.94%), 15A (*n* = 13, 4.94%) and 6E (*n* = 13, 4.94%). Detailed information can be found in Supplementary Table 1. The coverage rates for PCV10, PCV13, PCV15, PCV20 and PCV24 were 36.12% (*n* = 95), 61.98% (*n* = 163), 61.98% (*n* = 163), 63.12% (*n* = 166) and 64.26% (*n* = 169), respectively (Figure 1). The differences of the prevalent serotypes in this study and our previous report (Yan et al., 2019) was shown in Supplementary Figure 1 and Supplementary Table 2.

**Figure 2:**
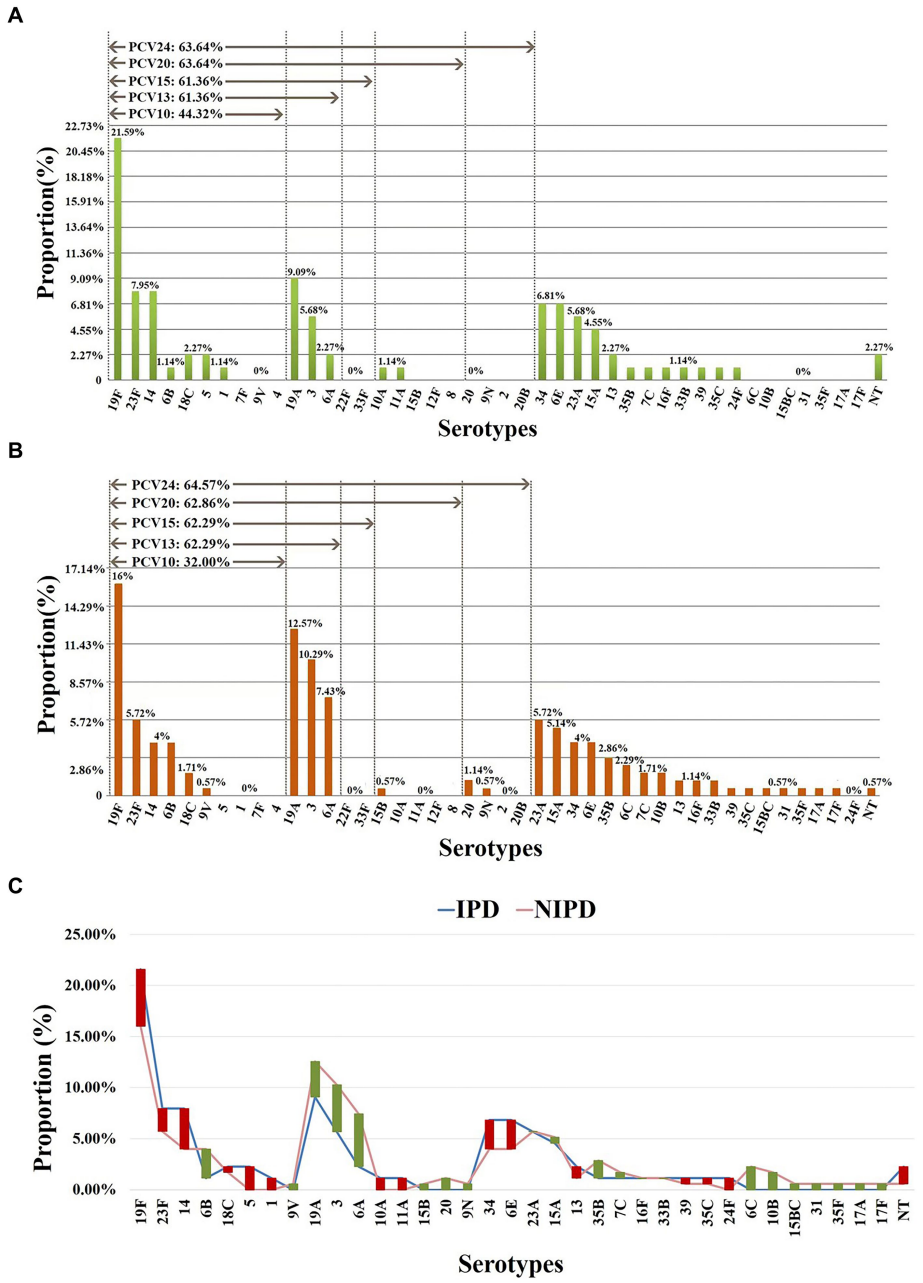
Serotype distribution in different diseases. **(A)** Proportions of each serotype in 88 IPD cases. **(B)** Proportions of each serotype in 175 NIPD cases. **(C)** The difference in the proportion of each serotype in IPD/NIPD cases. Red bar: the length indicates the value with a higher proportion of IPD than NIPD; green bar: the length indicates the value with a higher proportion of NIPD than IPD. NT, nontypable.

As classified by disease, 24 serotypes were identified in 88 IPD cases, and the prevalent serotypes in IPD were 19F (*n* = 19, 21.59%), 19A (*n* = 8, 9.09%), 14 (*n* = 7, 7.95%), 23F (*n* = 7, 7.95%), 34 (*n* = 6, 6.82%) and 6E (*n* = 6, 6.82%) (Supplementary Table 3), with coverage rates for PCV10, PCV13, PCV15, PCV20 and PCV24 of 44.32% (*n* = 39), 61.36% (*n* = 54), 61.36% (*n* = 54), 63.64% (*n* = 56) and 63.64% (*n* = 56), respectively (Figure 2). Thirty serotypes were identified in 175 NIPD cases, and the prevalent serotypes in NIPD were 19F (*n* = 28, 16.00%), 19A (*n* = 22, 12.57%), 3 (*n* = 18, 10.29%), 6A (*n* = 13, 7.43%), 23F (*n* = 10, 5.71%) and 23A (*n* = 10, 5.71%) (Supplementary Table 4), with coverage rates for PCV10, PCV13, PCV15, PCV20 and PCV24 of 32.00% (*n* = 56), 62.92% (*n* = 109), 62.92% (*n* = 109), 62.86% (*n* = 110) and 64.57% (*n* = 113), respectively (Figure 2).

**Figure 3:**
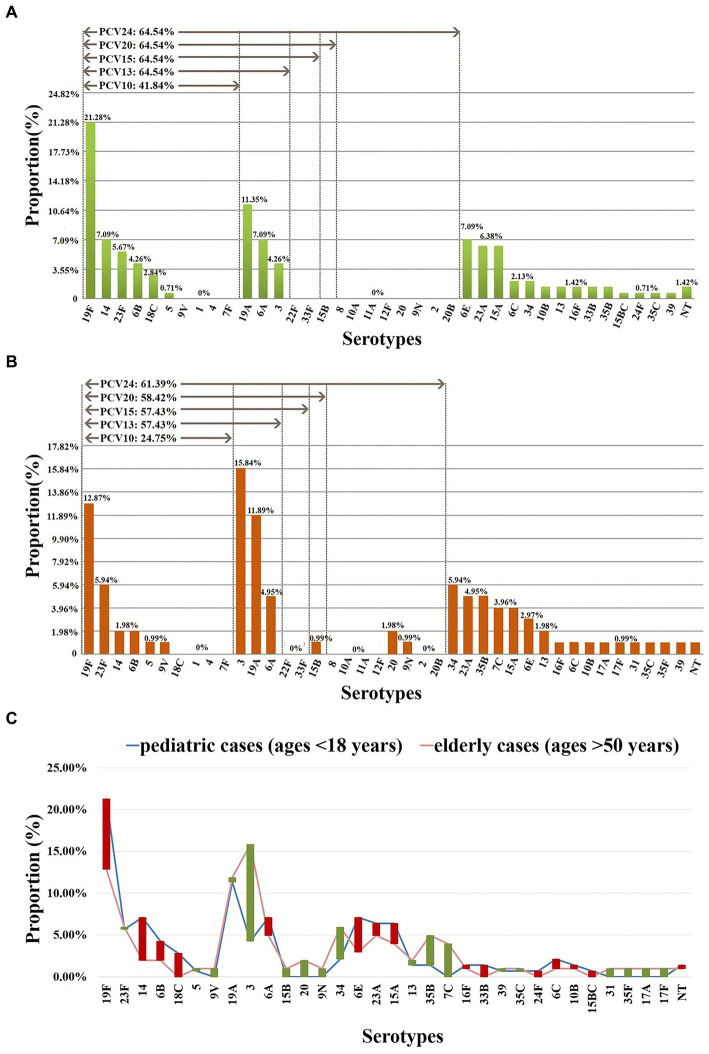
Serotype distribution in different ages. **(A)** Proportions of each serotype in 141 pediatric cases (ages <18 years). **(B)** Proportions of each serotype in 101 elderly cases (ages >50 years). **(C)** The difference in the proportion of each serotype in pediatric/elderly cases. Red bar: the length indicates the value with a higher proportion of pediatric cases than elderly cases; green bar: the length indicates the value with a higher proportion of elderly cases than pediatric cases. NT, nontypable. Proportions of each serotype in patients aged 18–50 years old were shown in Supplementary Table 7.

As classified by age, 23 serotypes were identified in 141 pediatric cases (age < 18 years); the prevalent serotypes were 19F (*n* = 30, 21.28%), 19A (*n* = 16, 11.35%), 6A (*n* = 10, 7.09%), 6E (*n* = 10, 7.09%), 14 (*n* = 10, 7.09%), 15A (*n* = 9, 6.38%), 23A (*n* = 9, 6.38%) (Supplementary Table 5), with coverage rates for PCV10 and PCV13/PCV15/PCV20/PCV24 of 41.84% (*n* = 59) and 64.54% (*n* = 91), respectively (Figure 3). In addition, 28 serotypes were identified in 101 elderly patients (age > 50 years); the prevalent serotypes were 3 (*n* = 16, 15.84%), 19F (*n* = 13, 12.87%), 19A (*n* = 12, 11.88%), 34 (*n* = 6, 5.94%), 23F (*n* = 6, 5.94%), 6A (*n* = 5, 4.95%), 23A (*n* = 5, 4.95%), 35B (*n* = 5, 4.95%) (Supplementary Table 6), with coverage rates for PCV10, PCV13, PCV15, PCV20 and PCV24 of 24.75% (*n* = 25), 57.43% (*n* = 58), 57.43% (*n* = 58), 58.42% (*n* = 59) and 61.39% (*n* = 62), respectively (Figure 3). The prevalent serotypes in patients aged 18–50 years old were shown in Supplementary Table 7.

### 3.3 Multilocus sequence typing

**Figure 4:**
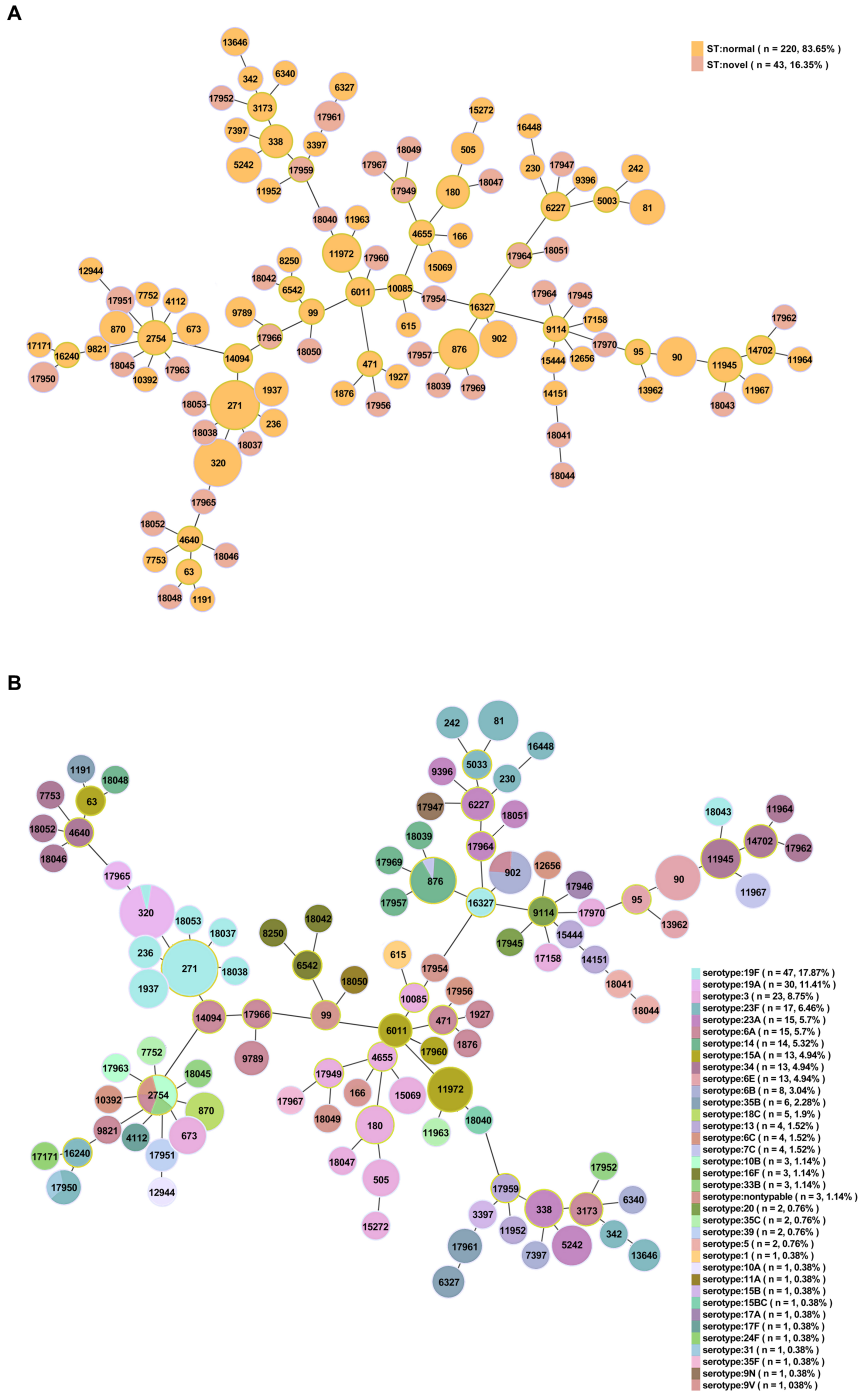
Minimum spanning tree-like structure via the goeBURST full MST algorithm (level 6). **(A)** Thirty-eight novel STs among 263 *S. pneumoniae* isolates (*n* = 43). Pink: novel STs. **(B)** The relationship between ST and serotype. Each disk represents an ST, and each color represents a serotype.

Among the 263 isolates, 107 different STs were successfully identified by MLST analysis, including 38 novel STs (*n* = 43/263, 35.51%): ST17945-ST17947, ST17949-ST17952, ST17954, ST17956-ST17957, ST17959-ST17967, ST17969, ST17970, and ST18037-ST18053 (Figure 4A). Additionally, 19 novel alleles were identified by sequencing: *aroE* (617), *ddl* (1163–1,165), *gdh* (798–800), *gki* (825–830), *recP* (563–565), *spi* (777, 778) and *xpt* (1087). The numbers of novel STs and alleles were assigned by the MLST database after the sequences were identified (see text footnote 2). Prevalent STs included ST271 (*n* = 33, 12.55%), ST320 (*n* = 31, 11.79%), ST90 (*n* = 11, 4.18%), ST876 (*n* = 11, 4.18%), ST11972 (*n* = 9, 3.42%), ST902 (*n* = 8, 3.04%), ST81 (*n* = 6, 2.28%) and ST5242 (*n* = 6, 2.28%); detailed information can be found in Supplementary Table 8.

Nine global clones were found in this study by comparing strains with the pneumococcal molecular epidemiology network (PMEN), including Spain^6B^-2 (ST90, *n* = 11, 4.2%), Spain^23F^-1 (ST81, *n* = 6, 2.3%), Netherlands^3^-31 (ST180, *n* = 4, 1.5%), Colombia^23F^-26 (ST338, *n* = 4, 1.5%), Taiwan^19F^-14 (ST236, *n* = 2, 0.8%), Taiwan^23F^-15 (ST242, *n* = 2, 0.8%), Sweden^15A^-25 (ST63, *n* = 1, 0.4%), USA^1^-29 (ST615, *n* = 1, 0.4%), and Denmark^14^-32 (ST230, *n* = 1, 0.4%).

**Figure 5:**
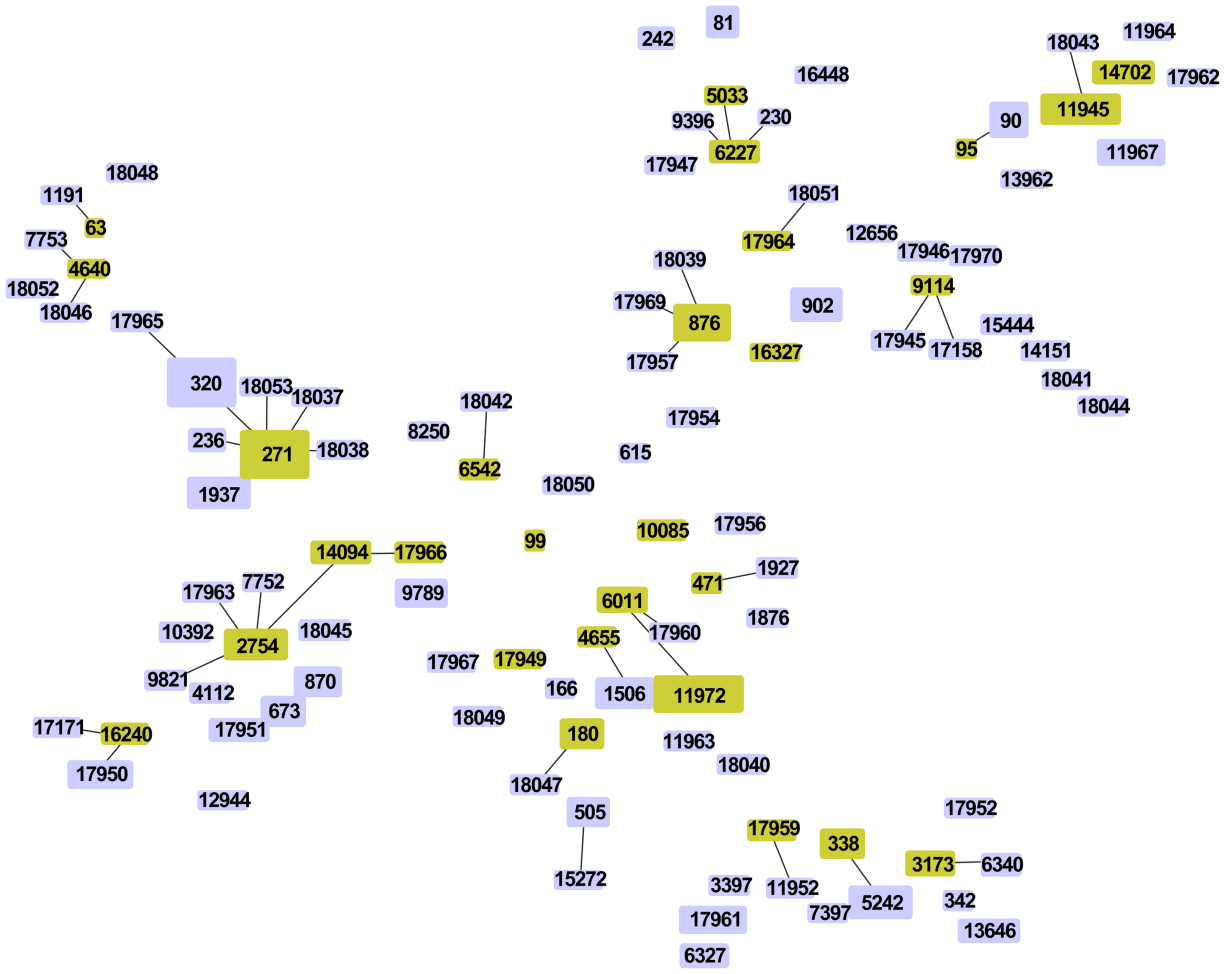
Minimum spanning tree-like structure via goeBURST distance analysis among 263 *S. pneumoniae* isolates (SLVs, level 1). SLV analysis showed that the 107 STs were divided into 21 CCs (linked) and 48 singletons.

Among the 263 isolates, 21 clonal complexes (CCs) and 48 singletons were obtained according to single-locus variants (SLVs) via goeBURST distance analysis (SLV, level 1, Figure 5). CC271 (*n* = 75, 28.5%), CC876 (*n* = 14, 5.3%), CC90 (*n* = 12, 4.6%), CC6011 (*n* = 12, 4.6%), CC2754 (*n* = 11, 4.2%) and CC338 (*n* = 10, 3.8%) were prevalent CCs.

**Figure 6:**
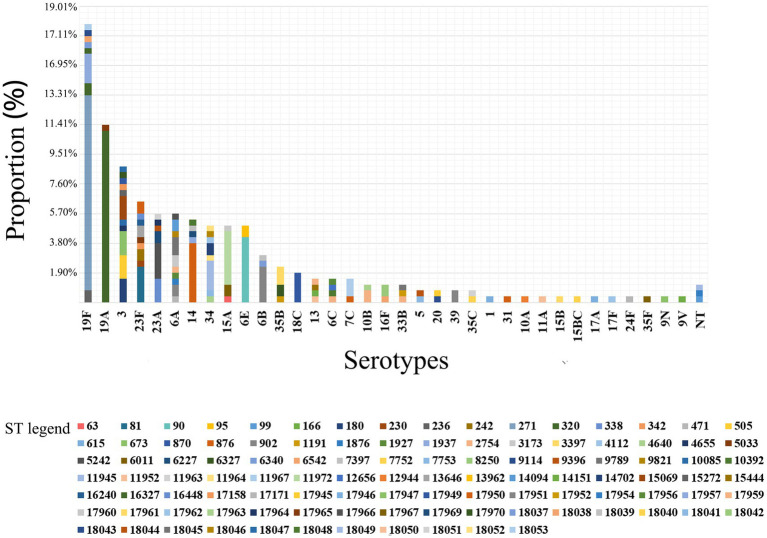
ST distribution in each serotype among 263 *S. pneumoniae* isolates. Serotype 19F was dominated by ST271, accounting for 70.21% (*n* = 33/47); serotype 19A was dominated by ST320, accounting for 96.67% (*n* = 29/30).

By comparing STs with serotypes, a minimum spanning tree-like structure was illustrated via the goeBURST Full MST algorithm, and the serotype distribution showed ST/CC aggregation (Figure 4B and Supplementary Table 9). Serotype 19F was dominated by ST271, accounting for 70.21% (*n* = 33/47); serotype 19A was dominated by ST320, accounting for 96.67% (*n* = 29/30); serotype 23F was dominated by ST81, accounting for 35.29% (*n* = 6/17); serotype 14 was dominated by ST876, accounting for 71.43% (*n* = 10/14); serotype 34 was dominated by ST11945, accounting for 38.46% (*n* = 5/13); serotype 15A was dominated by ST11972, accounting for 69.23% (*n* = 9/13); serotype 6E was dominated by ST90, accounting for 92.31% (*n* = 12/13); and serotype 6B was dominated by ST902, accounting for 75.00% (*n* = 6/8) (Figure 6). The serotypes of isolates among novel STs were distributed in 3, 5, 13, 14, 20, 31, 34, 39, 10B, 11A, 15A, 15 BC, 16F, 17A, 19A, 19F, 23A, 23F, 33B, 35B, 35F, 6A, 6C and 9 N.

**Figure 7:**
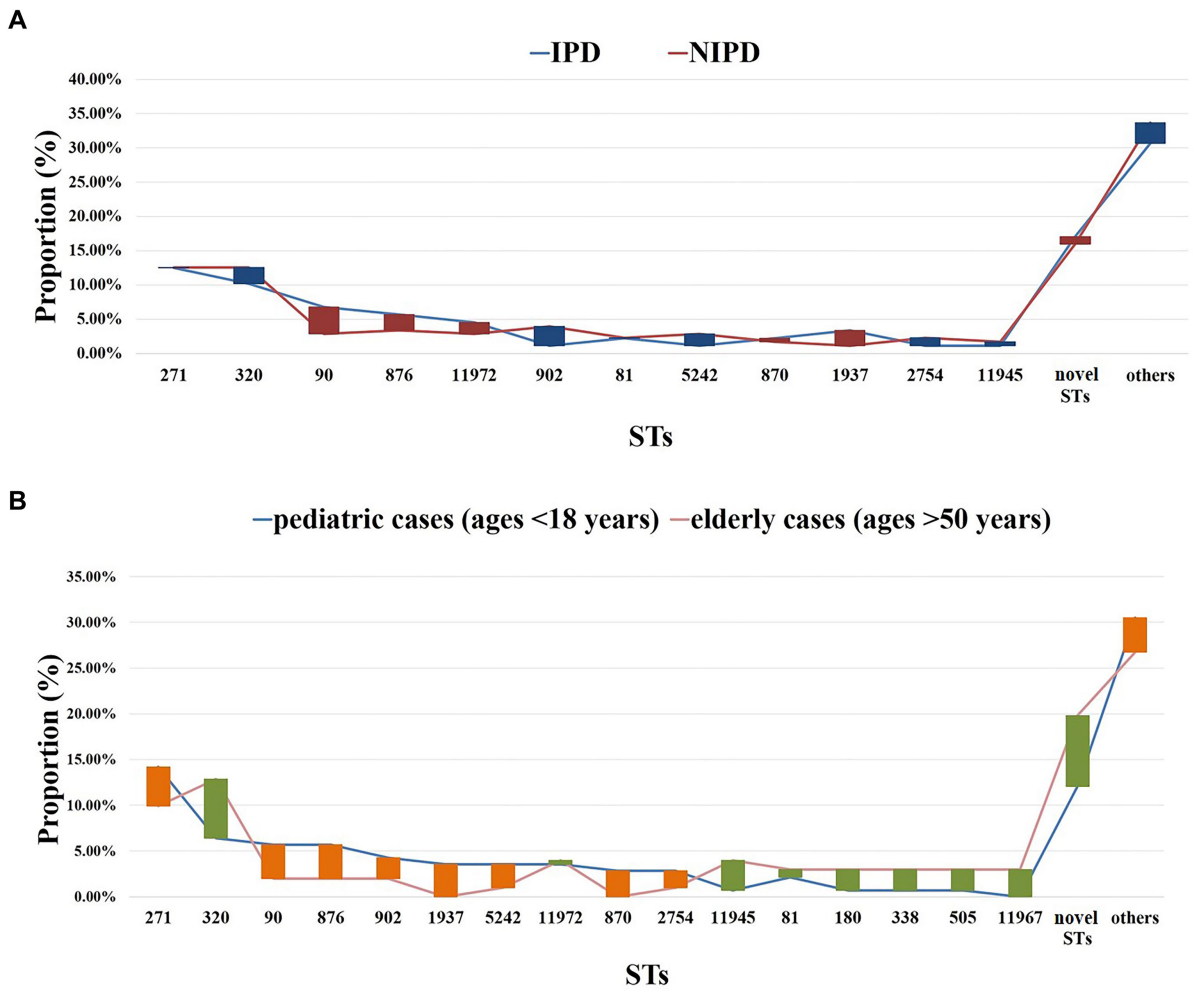
ST distribution in each group. **(A)** Proportions of each ST in different diseases. Blue line: IPD cases (*n* = 88); red line: NIPD cases (*n* = 175); red bar: the length indicates the value with a higher proportion of IPD than NIPD; blue bar: the length indicates the value with a higher proportion of NIPD than IPD. **(B)** Proportions of each ST in different ages. Blue line: pediatric cases (*n* = 141, ages <18 years); pink line: elderly cases (*n* = 101, ages >50 years); orange bar: the length indicates the value with a higher proportion of pediatric cases than elderly cases; green bar: the length indicates the value with a higher proportion of elderly cases than pediatric cases. Proportions of each ST in patients aged 18–50 years old were shown in Supplementary Table 7.

Regarding classification by disease, prevalent STs in IPD (88/263) were ST271 (*n* = 11, 12.50%), ST320 (*n* = 9, 10.23%), ST90 (*n* = 6, 6.82%), ST876 (*n* = 5, 5.68%), ST11972 (*n* = 4, 4.55%) and ST1937 (*n* = 3, 3.41%). Prevalent STs in NIPD (175/263) were ST271 (*n* = 22, 12.57%), ST320 (*n* = 22, 12.57%), ST902 (*n* = 7, 4.00%), ST876 (*n* = 6, 3.43%), ST90 (*n* = 5, 2.86%), ST5242 (*n* = 5, 2.86%), ST11972 (*n* = 5, 2.86%), ST81 (*n* = 4, 2.29%) and ST2754 (*n* = 4, 2.29%) (Figure 7A).

When classified by age, prevalent STs in pediatric cases (age < 18 years, *n* = 141/263) were ST271 (*n* = 20, 14.18%), ST320 (*n* = 9, 6.38%), ST90 (*n* = 8, 5.67%), ST876 (*n* = 8, 5.67%), ST902 (*n* = 6, 4.26%), ST1937 (*n* = 5, 3.55%), ST5242 (*n* = 5, 3.55%) and ST11972 (*n* = 5, 3.55%). Prevalent STs in elderly patients (age > 50 years) were ST271 (*n* = 10, 9.9%), ST320 (*n* = 13, 12.87%), ST11945 (*n* = 4, 3.96%), ST11972 (*n* = 4, 3.96%), ST81 (*n* = 3, 2.97%), ST180 (*n* = 3, 2.97%), ST338 (*n* = 3, 2.97%), ST505 (*n* = 3, 2.97%) and ST11967 (*n* = 3, 2.97%) (Figure 7B).

### 3.4 Phylogenetic analysis

**Figure 8:**
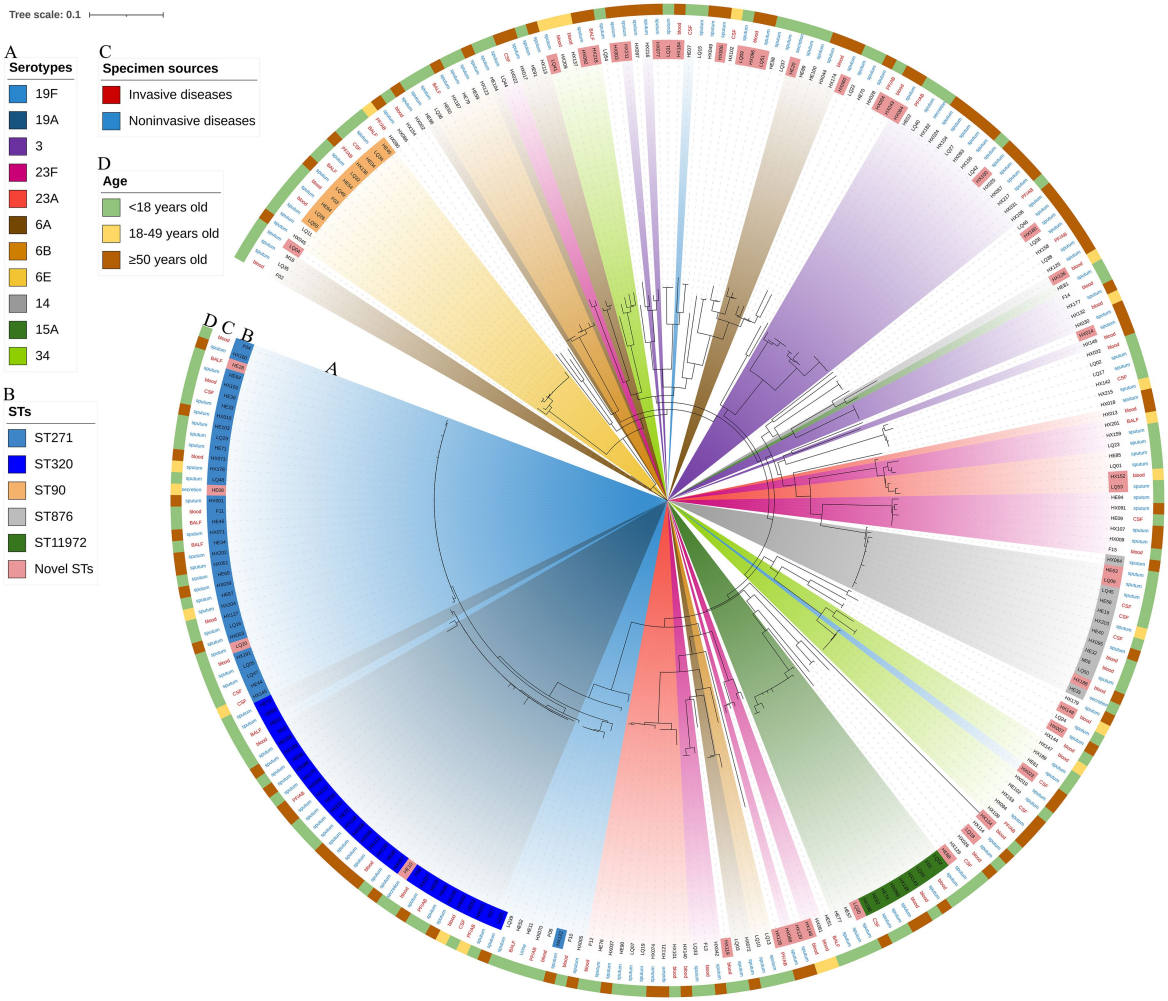
Maximum likelihood tree of 263 *S. pneumoniae* isolates. The RAxML tree was constructed from a multiFASTA alignment of core genes (>99%) using the GTRGAMMA method. **(A)** Serotypes with a proportion greater than 3%. **(B)** The top 5 proportion STs and novel STs. **(C)** Specimen sources (colored with IPD and NIPD). **(D)** Patients ages.

The RAxML tree based on 263 isolate core gene sequences was visualized and annotated with strain, ST, serotype, age, specimen source and disease. Detailed information is shown in Figure 8.

### 3.5 Antibiotic susceptibility

The overall prevalence of PEN-nonsusceptible *S. pneumoniae* (PNSP) was 6.1% in nonmeningitis cases, including PEN-intermediate *S. pneumoniae* (PISP, 4.88%) and PEN-resistant *S. pneumoniae* (PRSP, 1.22%), and PRSP in meningitis cases was 82.35%. Most isolates showed high resistance to ERY (96.96%) and TET (79.85%), and more than half of the isolates were resistant to SXT (58.94%). A few isolates were resistant to AMX (9.89%), CTX (11.03%), MEN (9.13%), OFX (1.14%), LVX (1.14%) and MXF (0.38%). However, no isolate was found to have VAN- and LNZ-resistant phenotypes. The detailed information is provided in Supplementary Figure 2 and Supplementary Table 10.

The relationship between antibiotic susceptibility and serotype is shown in Supplementary Figure 3A and Supplementary Table 11. In brief, serotypes 19A and 19F tended to be more resistant to AMX, CTX, MEM and SXT, whereas serotype 3 was more sensitive to these antibiotics. In addition, serotypes covered by PCV13 and PCV23 showed more resistance to PEN, AMX, CTX and SXT (Table 2). The differences of the antibiotic resistance in this study and our previous report (Yan et al., 2019) was shown in Supplementary Table 12.

**Table 2:** Relationship between antibiotic resistance and vaccine coverage.

Antibiotic		R%		R%			
		PCV13	Non-PCV13	*p*	PPV23	Non-PPV23	*p*
		(*n* = 163)	(*n* = 100)		(*n* = 155)	(*n* = 108)	
PEN	(meningitis)	91.67	60.00	0.450	84.62	75.00	0.154
	(nonmeningitis)	0.61	2.00	0.034	0.65	1.85	0.031
AMX		14.11	3.00	0.007	14.84	2.78	0.000
CTX		14.72	5.00	0.015	16.13	3.70	0.002
MEM		12.27	4.00	0.051	12.90	3.70	0.016
VAN		0.00	0.00	NA	0.00	0.00	NA
ERY		96.93	97.00	1.000	96.77	97.22	1.000
OFX		0.00	3.00	0.000	0.00	2.78	0.001
LVX		0.00	3.00	0.053	0.00	2.78	0.062
MFX		0.00	1.00	0.617	0.00	0.93	0.636
TET		80.37	79.00	0.894	81.29	77.78	0.639
SXT		65.03	49.00	0.009	65.16	50.00	0.008
LNZ		0.00	0.00	NA	0.00	0.00	NA

The relationship between antibiotic susceptibility and STs is indicated in Supplementary Figure 3B and Supplementary Table 13. In brief, ST271 tended to be more resistant to AMX and CTX, whereas ST90 was more sensitive to AMX, CTX and MEN. ST876 and ST11972 were more sensitive to AMX and ST902 to AMX, CTX, MEN and SXT.

Among 263 isolates, 53.61% (*n* = 141) were defined as multidrug resistant (MDR), leading to serotypes 19F (*n* = 36, 25.35%), 19A (*n* = 26, 18.31%), ST271 (*n* = 28, 19.72%) and ST320 (*n* = 27, 19.01%). In addition, isolates with serotypes covered by PCV13 accounted for 70.21% (*n* = 99) of MDR isolates. Among MDR isolates, the most common phenotype was ERY/TET/SXT (*n* = 78/142, 54.9%), including 22 IPD cases and 56 NIPD cases, with the leading serotypes of 19A (*n* = 12, 15.38%), 19F (*n* = 9, 11.54%), 23F (*n* = 8, 10.26%), and 6E (*n* = 8, 10.26%) and leading STs of ST320 (*n* = 12, 15.38%), ST90 (*n* = 7, 8.94%), ST271 (*n* = 5, 6.41%), and ST870 (*n* = 4, 5.13%). Other MDR phenotypes included CTX/ERY/TET/SXT (*n* = 20/142, 14.1%), with the leading serotype being 19F (*n* = 16, 80.00%) and leading ST being ST271 (*n* = 13, 65.00%), and MEM/ERY/TET/SXT (*n* = 17/142, 12.0%), with leading serotypes 19F (*n* = 7, 41.18%) and 19A (*n* = 6, 35.29%) and leading STs ST271 (*n* = 6, 35.29%) and ST320 (*n* = 6, 35.29%).

### 3.6 Resistance gene analysis

**Figure 9:**
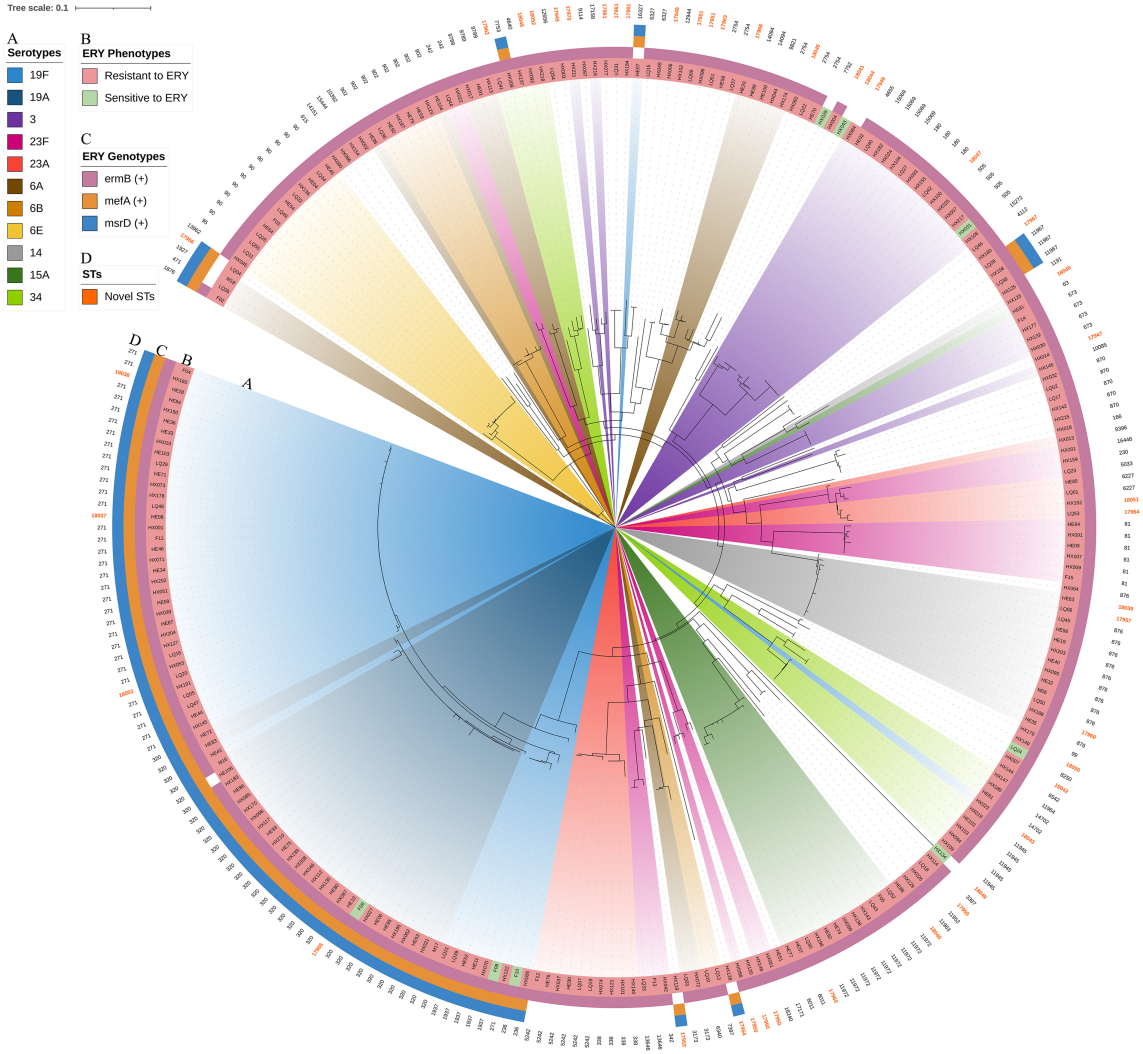
The relationship among erythromycin resistance phenotypes, erythromycin resistance genes, STs and serotypes. The RAxML tree was annotated with strain number, serotypes, erythromycin resistance phenotypes, erythromycin resistance genes (*msrD*, *MefA* and *ermB*) and STs. Erythromycin resistance genes manifested as an aggregation phenomenon. **(A)** Serotypes with a proportion greater than 3%. **(B)** Erythromycin resistance phenotypes. **(C)** Erythromycin resistance genes. **(D)** Novel STs were colored.

**Figure 10:**
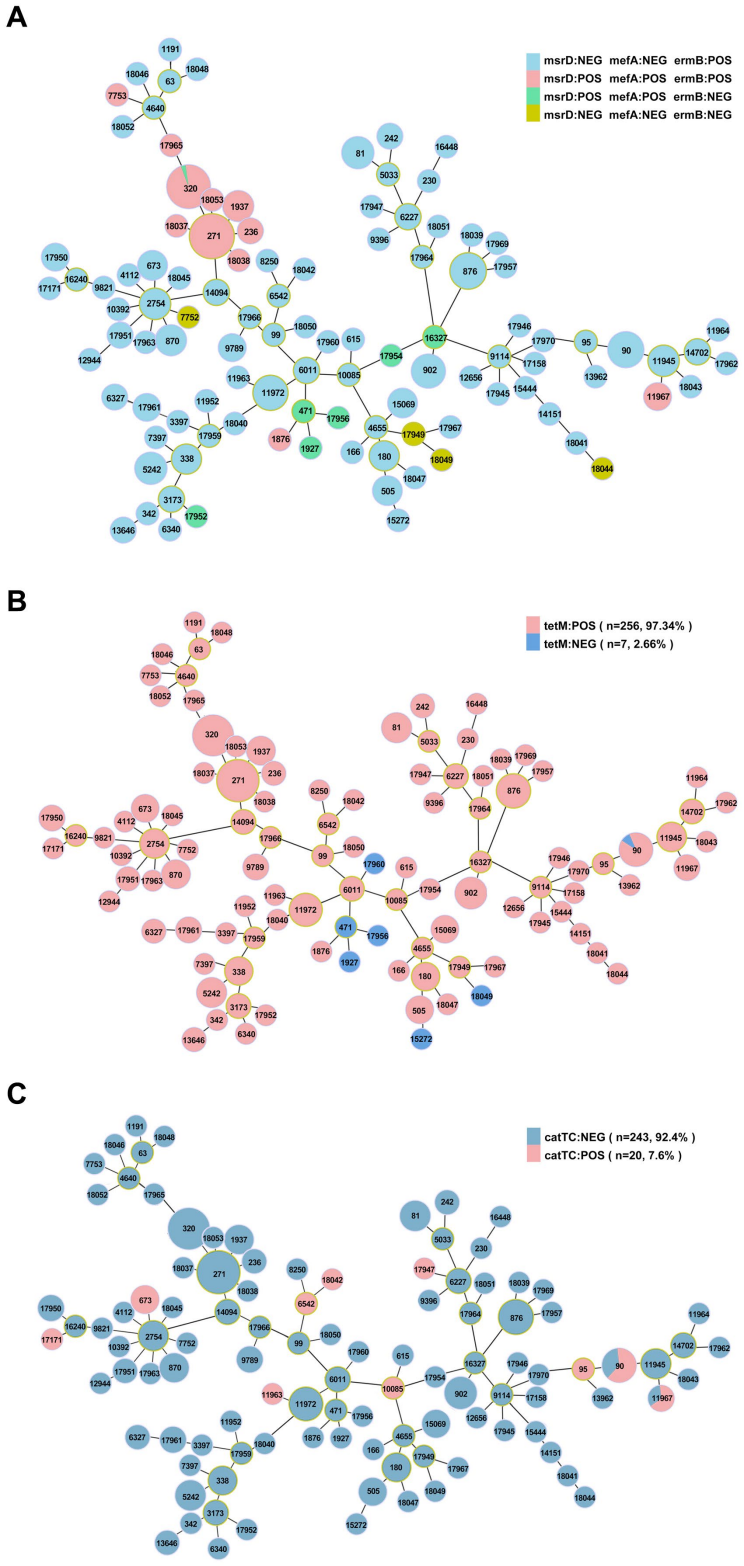
The relationship between antibiotic resistance genes and ST. **(A)** Minimum spanning tree-like structure showing the ST aggregation phenomenon of the erythromycin resistance genes *msrD*, *MefA* and *ermB.* **(B)** The relationship between the tetracycline resistance gene *tetM* and ST. **(C)** The relationship between the chloramphenicol resistance gene *catTC* and ST.

The resistance genes *msrD* (*n* = 86, 32.7%)*, mefA* (*n* = 86, 32.7%)*, ermB* (*n* = 252, 95.8%)*, tetM* (*n* = 256, 97.3%) and *catTC* (*n* = 20, 7.6%) were found among the 263 isolates, which are associated with erythromycin, tetracycline and chloramphenicol. The erythromycin resistance genes *msrD*, *mefA* and *ermB* manifested as ST aggregation phenomena, and 28.9% (*n* = 76) of the isolates carried these three resistance genes, including ST271 (*n* = 33/76, 43.4%) and ST320 (*n* = 29/76, 38.2%) (Figures 9, 10). However, the ST aggregation of the tetracycline resistance gene *tetM* and chloramphenicol resistance gene *catTC* was not significant (Figure 10).

## 4 Discussion

In this study, we collected *S. pneumoniae* isolates from 263 PD patients in 5 hospitals and performed whole-genome sequencing. To our knowledge, this is the first all-age multicenter *S. pneumoniae* study conducted in Southwest China. Comprehensive identification of the molecular characteristics of pneumococcal isolates revealed prevalence characteristics, including serotype, ST, and the relationship among antibiotic susceptibility and the carrying antibiotic resistance genes in Southwest China from 2018 to 2022, covering the period before and during the COVID-19 outbreak. The results showed that 19F, 19A, 3, 23F, 6A, 23A, 14, 34, 15A and 6E were the top 10 prevalent serotypes. ST271, ST320, ST90, ST876, ST11972, ST902, ST81 and ST5242 were the most prevalent STs, and CC271, CC876, CC90, CC6011, CC2754 and CC338 were the most common CCs. Most isolates were resistant to ERY (96.96%) and TET (79.85%), and more than half of the isolates were resistant to SXT (58.94%).

The clinical isolates of *S. pneumoniae* were highly serotype heterogeneous. We identified 35 different serotypes, and 3 isolates were untypable. The top 5 serotypes in prevalence (19F, 19A, 3, 23F, and 6A) accounted for 50.19%, which was slightly different from the results reported previously in this region among pediatric patients (19F, 19A, 6B, 6A, and 14) (Yan et al., 2021), as well as those reported in Shanghai (19F, 6A, 19A, 23F and 14) (Zhao et al., 2019), Chongqing (19F, 6A/B, 19A) (Yu et al., 2019) and Beijing (19F, 19A, 23F, 14 and 6A) (Wang Q. et al., 2020). In this study, the most common serotypes among 88 IPD strains were 19F, 19A, 14, 23F, 34 and 6E. These findings are similar to the results of a multicenter study conducted in China (Zhao et al., 2013). Among 175 NIPD strains, the most common serotypes were 19F, 19A, 3, 6A, 23F and 23A. The prevalence of serotypes 3 and 23F in elderly patients was significantly higher than that in children. This finding, primarily the result of serotype 3, is significantly higher than our previous findings (8.75% vs. 3.13%) (Yan et al., 2021). The increase in the prevalence of serotype 3 is similar to the prevalence of *S. pneumoniae* in some developed countries (Goettler et al., 2020; Suaya et al., 2020; Hyams et al., 2023). Compared with our previous study (Yan et al., 2021), the PCV-covered serotypes decreased slightly (PCV10: 50.8% vs. 36.12%; PCV13: 77.3% vs. 61.98%). The difference in serotype distribution of *S. pneumoniae* may be due to serotype replacement after widespread use of PCVs (Hanquet et al., 2022; Lo et al., 2022).

We analyzed 263 isolates by MLST, and the results showed that ST271, ST320, ST90 and ST876 were the most prevalent STs in Southwest China, similar to other parts in China (Wang X. et al., 2020; Zhou et al., 2022) but different from those in other countries, such as northern Russia (Vorobieva S Jensen et al., 2020) and Latin America (Moreno et al., 2020). The present study further revealed 38 novel STs, which accounted for 35.51% of all isolates (43/263), strongly indicating the global diversity of *S. pneumoniae*. In addition, the study identified 19 novel alleles through sequencing. Phylogenetic tree analysis showed CC271, CC876, CC90, CC6011, and CC2754 to be common CCs, among which CC271 is prevalent globally and the most common CC in China (Zeng et al., 2023).

Penicillin has been the first-line treatment for *S. pneumoniae*, especially before the 1980s (Swartz, 2002). The current work shows that the resistance rate of isolates to PEN was 1.22% in nonmeningitis patients, consistent with results reported from other parts of East Asia, such as China (0.7–2.2%), Korea (1.0%) and Japan (1.7–2.2%) (Wang et al., 2019; Kim et al., 2020; Tsuzuki et al., 2020). These results suggest that PEN can continue to be used in treatment against nonmeningitis *S. pneumoniae* at higher concentrations but not against meningitis strains.

Macrolides and fluoroquinolones are the main alternatives to beta-lactam antibiotics in cases of reduced susceptibility to *S. pneumoniae* infections (Berbel et al., 2022). However, with widespread clinical use of macrolide antibacterial agents, the number of ERY-resistant *S. pneumoniae* (ERSP) strains has increased. This phenomenon is a cause for concern, and macrolides have been included in the list of Critically Important Antimicrobials for Human Medicine by the WHO. According to data from the China Antimicrobial Resistance Surveillance System in 2021, the resistance rate of *S. pneumoniae* to ERY average as high as 96.4% nationwide. In this study, rates of macrolide resistance (MR) were significantly high, with a rate of resistance to ERY of 96.96%, indicating that these agents had little clinical utility for treatment of *S. pneumoniae* infections, which is consistent with the results in Northeast China (98.57%) (Zhou et al., 2022) and Beijing (96.4%) (Zhou et al., 2012).

In China, MR increased from 15 to 95% over 16 years (2000–2016), mainly associated with the spread of *ermB* (Ip et al., 2002; Lu et al., 2017). Determinants of MR include modification by ribosomal methylases (*ermB*) and active antibiotic efflux pumps (*mefA*/*E*). *ermB* is the most common mechanism of ERY resistance in Asia, including mainland China, Taiwan, Sri Lanka, South Korea and Japan, whereas *mefA* is more common in Hong Kong, Singapore, Malaysia, India and the Philippines (Song et al., 2004; Kim et al., 2012). This is consistent with the results for *ermB* (252/263) and *mefA* (86/263) in this study, which may be responsible for the high degree of ERY resistance in this region. In Europe, *ermB* was found to be more common in Italy (55.8%), Poland (80.8%), Serbia (82.4%), Spain (88.3%), France (90%) and Belgium (91.5%) (Felmingham et al., 2007); furthermore, *mefA* is common in Germany (53.2%), Finland (55.4%), Australia (59.5%), Greece (66.2%) and the United Kingdom (70.8%) (Farrell et al., 2008).

Beta-lactam antibiotics and macrolides are commonly used to treat community-acquired infections in children because they are relatively safe and inexpensive. This chronic antibiotic pressure also contributes to resistance. In contrast, tetracyclines, quinolones and sulfonamides are prohibited or restricted in children of different ages due to their toxicity and adverse effects.

The resistance of *S. pneumoniae* to TET is also notably high in China, which may be related to misuse of TET in agriculture and livestock (Zhou et al., 2012). *tetM*, encoding ribosome protection proteins, is one of the causes of TET resistance (Widdowson et al., 1996; Widdowson and Klugman, 1998). In this study, the rate of TET resistance was 79.85%, whereas *tetM* was detected in almost all TET-resistant strains.

Development of resistance to any three or more antimicrobial agents of different classes is described as MDR. More than 30% of *S. pneumoniae* strains globally are reported to exhibit MDR (Jung et al., 2013). In a 2012–2017 study conducted in 6 Asian countries, 50.8% of *S. pneumoniae* strains exhibited MDR, with the highest rates observed in China (76.0%) and Korea (64.0%) (Kim et al., 2020), much higher than that in other regions of the world, such as 40.8% in France and 42.9% in Greece (Niedzielski et al., 2013). Singapore (17.9%) and the Philippines (3.1%) have the lowest rates in 6 Asian countries (Kim et al., 2020). In this study, the rate of MDR was 53.99% (141/263). MDR *S. pneumoniae* is a growing concern because the infecting strains are resistant to standard treatments with beta-lactams and macrolides.

In this study, several associations were found between serotype, ST, and antibiotic susceptibility. The dominant STs of serotype 19F, 19A, 23F and 14 isolates were ST271, ST320, ST81, and ST876, respectively, which was similar to previously reported results (Li et al., 2018). Isolates 19F/ST271 and 19A/ST320, as the predominant prevalent strains in this region, were more resistant to AMX, CTX, MEM and SXT. In terms of the distribution of antibiotic resistance among serotypes, serotypes 19F, 19A and 14 exhibited higher resistance rates, while serotype 3 showed a relatively low rate. The high level of resistance among serotypes may be related to widespread international spread of their STs. In this study, serotype 3 had a high clonal diversity, as dominated by ST180 (17.39%), ST505 (17.39%), ST15069 (17.39%) and ST180 (17.39%). A study that performed whole-genome sequencing of 616 strains of serotype 3 from England and Wales found that the composition of their clade changed with antibiotic resistance (Groves et al., 2019). These findings confirm the importance and utility of routine whole-genome sequencing and its ability to identify novel variants, which provides a basis for surveillance and will influence future vaccine development.

Children and the elderly are most vulnerable to *S. pneumoniae* infection. A global study of disease burden showed that infectious syndromes caused by bacterial pathogens pose an enormous threat to public health, with *S. pneumoniae* ranking among the top (Ikuta et al., 2022). Our study is the first to perform whole-genome sequencing of *S. pneumoniae* from PD patients of all ages in multiple centers in Southwest China. The total area of the southwestern region reaches 2.3406 million square kilometers, accounting for 24.5% of China’s land area. Its population and area are approximately half of those of the European Union.^12^ Thus, these research results are representative of the prevalence of *S. pneumoniae* in China. We discovered many novel ST types, and the whole-genome data can provide a basis for future research. In addition, the study incorporated data during the COVID-19 pandemic. As COVID-19 containment policies likely affected the spread of respiratory pathogens such as *S. pneumoniae* (Brueggemann et al., 2021), the molecular characterization in this study provides important support for the current status of PD and potential changes in subsequent isolates. However, it also has two limitations. First, this is a study in Southwest China and lacks research data from other regions. Second, our analysis of whole-genome data was not absolutely sufficient, prompting us to conduct more in-depth and correlation analyses in the future.

Overall, our study provides valuable insight into the prevalence and antibiotic susceptibility of PD-causing *S. pneumoniae* strains in Southwest China. The most prevalent strains in this study were 19F, 19A, 3, 23F, 6A and 23A; the prevalent STs were ST271, ST320, ST90, ST876 and ST11972. All strains were susceptible to VAN and LNZ but highly resistant to macrolides (96.96%), and the high prevalence of MR and MDR is alarming. These data highlight the importance of appropriate use of antimicrobial agents and underscore the need to monitor pneumococcal epidemiology in China. Considering the relatively high coverage rate of PCV13 and the worrisome rates of antibiotic nonsusceptibility, PCV13 vaccination may be beneficial in this region.

## Data availability statement

The datasets presented in this study are deposited in the MLST repository (https://pubmlst.org/bigsdb?db=pubmlst_spneumoniae_seqdef&page=query) under accession numbers: ST17945-ST17947, ST17949-ST17952, ST17954, ST17956-ST17957, ST17959-ST17967, ST17969, ST17970, and ST18037-ST18053.

## Ethics statement

The studies involving humans were approved by the Clinical Trial Ethics Committee of West China Second University Hospital, Sichuan University. The studies were conducted in accordance with the local legislation and institutional requirements. The human samples used in this study were acquired from primarily isolated as part of your previous study for which ethical approval was obtained. Written informed consent for participation was not required from the participants or the participants’ legal guardians/next of kin in accordance with the national legislation and institutional requirements.

## Author contributions

CM: Data curation, Investigation, Software, Writing – original draft. ZY: Software, Writing – original draft, Funding acquisition, Methodology. CC: Software, Writing – original draft, Visualization. LK: Writing – original draft, Validation. KA: Validation, Writing – original draft. YL: Writing – original draft, Data curation. JL: Writing – original draft, Validation. XH: Writing – original draft, Investigation. XZ: Investigation, Writing – original draft. YZ: Writing – original draft, Visualization. YC: Funding acquisition, Methodology, Resources, Writing – review & editing. YJ: Funding acquisition, Resources, Writing – review & editing, Conceptualization, Supervision. YX: Conceptualization, Resources, Supervision, Writing – review & editing.

## Footnotes

^1^ https://www.iso.org/standard/68763.html

^2^ https://pubmlst.org/

^3^ https://submit.ncbi.nlm.nih.gov/

^4^ https://pubmlst.org/organisms/streptococcus-pneumoniae

^5^ https://github.com/tseemann/mlst

^6^ http://www.phyloviz.net

^7^ https://github.com/sanger-pathogens/Roary

^8^ https://github.com/andrewjpage/snp-sites

^9^ https://github.com/stamatak/standard-RAxML

^10^ https://itol.embl.de

^11^ https://github.com/tseemann/abricate

^12^ https://ec.europa.eu/eurostat/web/main/home
