# Supplementary material for: Serotype, antibiotic susceptibility and whole-genome characterization of Streptococcus pneumoniae in all age groups living in Southwest China during 2018–2022
Source: Front Microbiol. 2024 Feb 1;15:1342839. doi: 10.3389/fmicb.2024.1342839 (PMC10867222; doi:10.3389/fmicb.2024.1342839)
Supplement: Supplementary file 1 [file Data_Sheet_1.pdf]

Supplementary Figure 1. Relationships of the top five serotypes between previous report (Yan et al., 2021) and current report. The results of the current report are divided into three groups according to age, namely, < 18 years old, 18-50 years old, and > 50 years old.

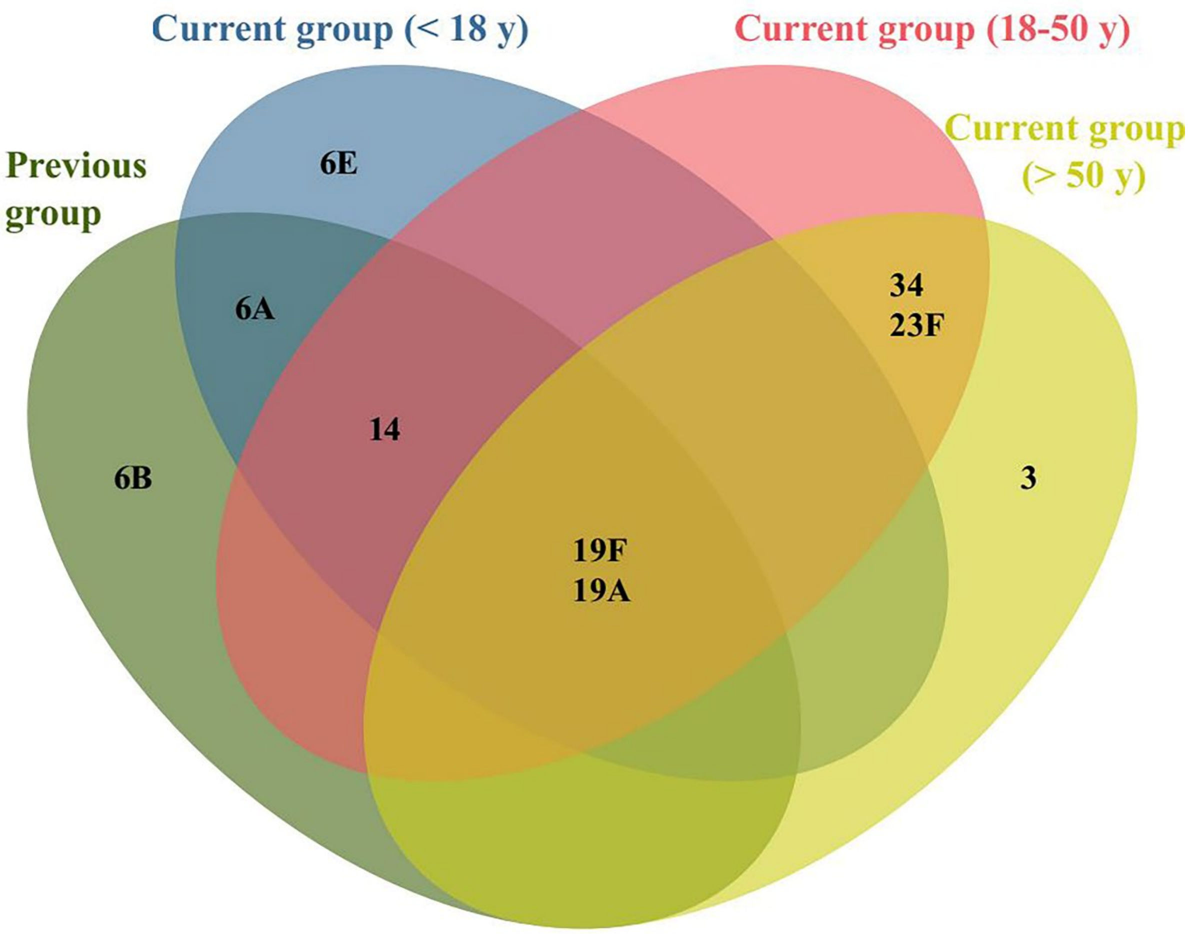

Supplementary Figure 2. Antibiotic resistance of 263 *S. pneumoniae* isolates. S: Sensitive, I: Intermediary, R: Resistant.

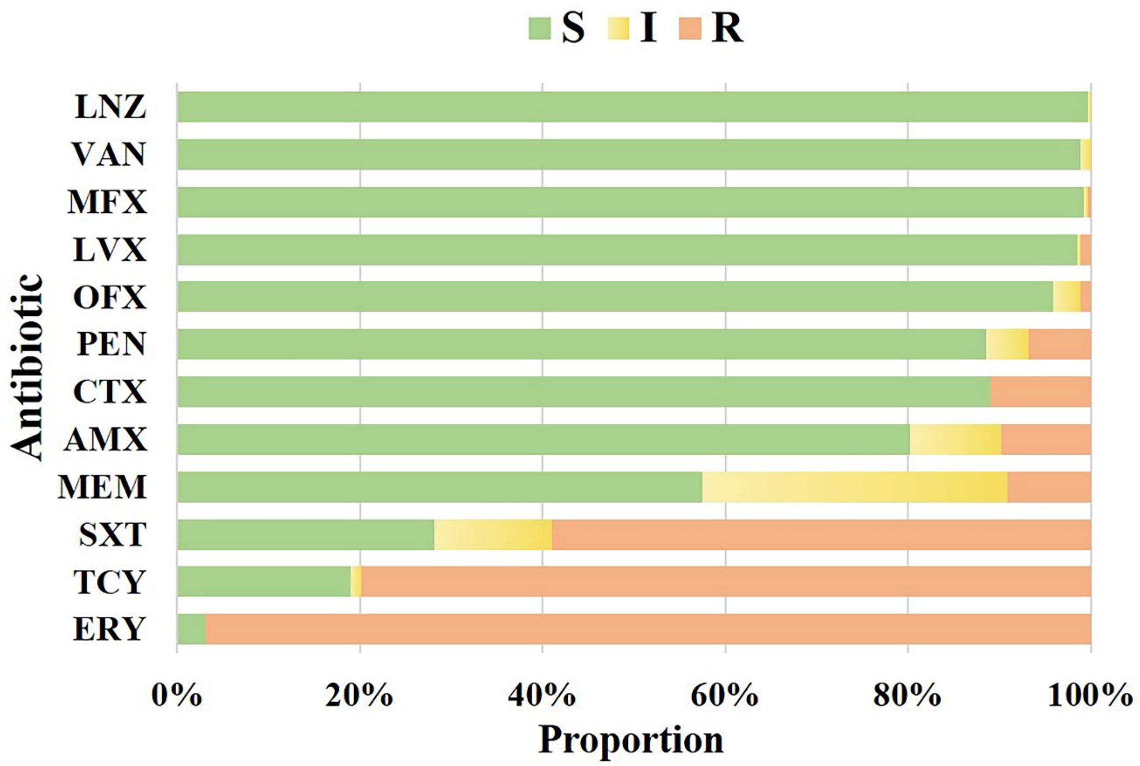

Supplementary Figure 3. Relationship between antibiotic resistance and serotype/ST. "*" represents a significant difference ($P<0.05$); "ns" means there is no significant difference. **(A)** Relationship between antibiotic resistance and serotypes. **(B)** Relationship between antibiotic resistance and STs.

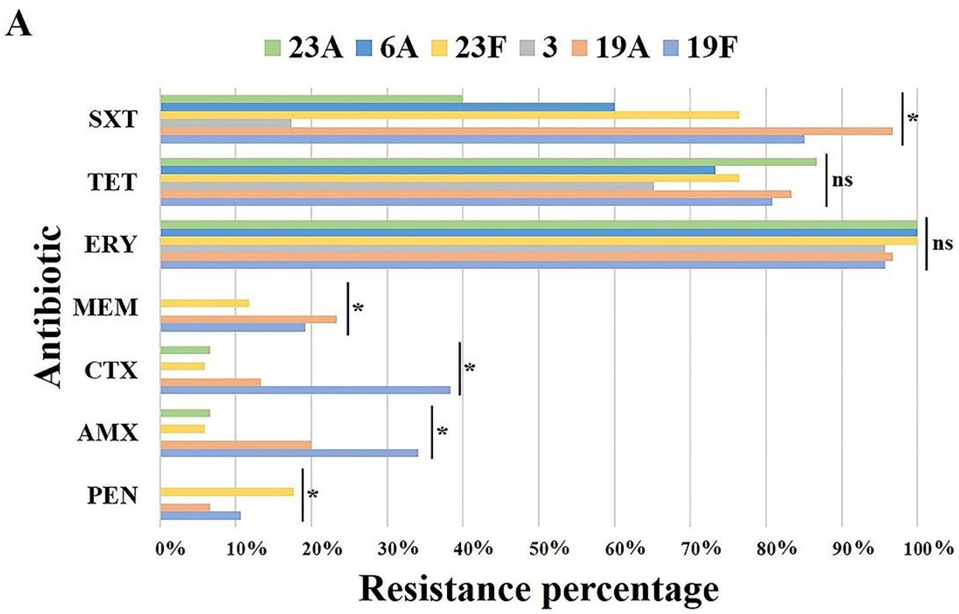

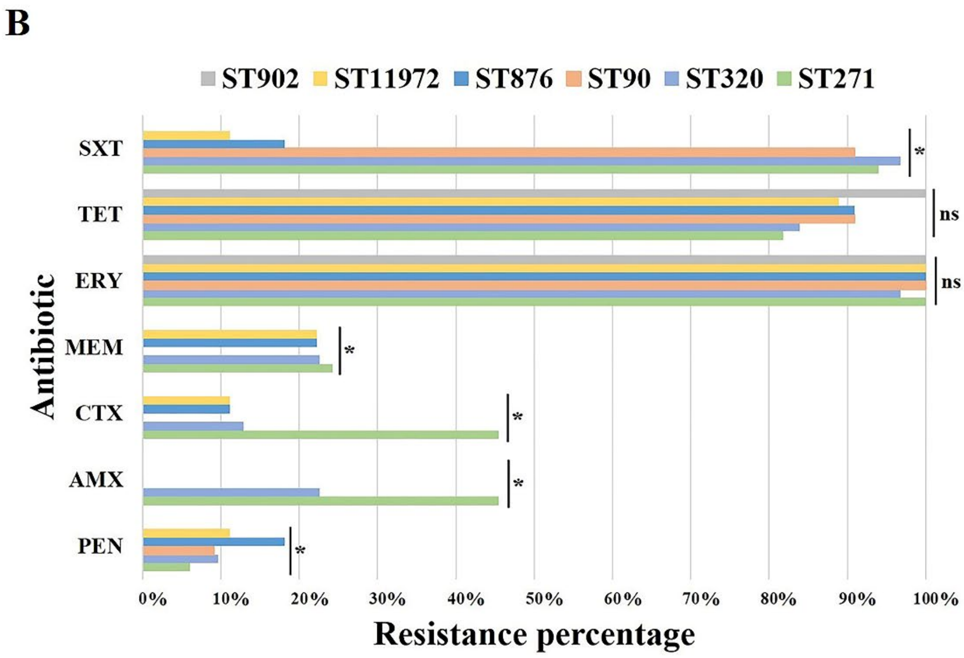

Supplementary Table 1. Serotype distribution among 263 *S. pneumoniae* isolates.

Serial number	Serotype	No. of isolates	Constituent ratio
1	19F	47	17.87%
2	19A	30	11.41%
3	3	23	8.75%
4	23F	17	6.46%
5	6A	15	5.70%
6	23A	15	5.70%
7	14	14	5.32%
8	34	13	4.94%
9	15A	13	4.94%
10	6E	13	4.94%
11	6B	8	3.04%
12	35B	6	2.28%
13	18C	5	1.90%
14	6C	4	1.52%
15	13	4	1.52%
16	7C	4	1.52%
17	16F	3	1.14%
18	10B	3	1.14%
19	33B	3	1.14%
20	20	2	0.76%
21	5	2	0.76%
22	39	2	0.76%
23	35C	2	0.76%
24	9V	1	0.38%
25	9N	1	0.38%
26	15B	1	0.38%
27	15BC	1	0.38%
28	1	1	0.38%
29	24F	1	0.38%
30	31	1	0.38%
31	11A	1	0.38%
32	35F	1	0.38%
33	10A	1	0.38%
34	17A	1	0.38%
35	17F	1	0.38%
36	Non-typable	3	1.14%
Total		263	100.00%

Supplementary Table 2. Results comparison of prevalent serotypes between the previous report (Yan et al., 2021) and the current report.

Serotypes	Previous report	Current report	*P*
19F	25.78**%** (33/128)	17.87% (47/263)	0.069
19A	14.06**%** (18/128)	11.41% (30/263)	0.453
**3**	**3.13% (4/128)**	**8.75% (23/263)**	**0.040**
23F	2.34**%** (3/128)	6.46% (17/263)	0.083
6A	9.38% (12/128)	5.70% (15/263)	0.152
23A	3.13% (4/128)	5.70% (15/263)	0.266
6B	3.91% (5/128)	3.04% (8/263)	0.765
14	7.81% (10/128)	5.32% (14/263)	0.336

Supplementary Table 3. Serotype distribution among 88 IPD cases.

Serial number	Serotype	No. of isolates	Constituent ratio
1	19F	19	21.59%
2	19A	8	9.09%
3	14	7	7.95%
4	23F	7	7.95%
5	34	6	6.82%
6	6E	6	6.82%
7	3	5	5.68%
8	23A	5	5.68%
9	15A	4	4.55%
10	13	2	2.27%
11	6A	2	2.27%
12	18C	2	2.27%
13	1	1	1.14%
14	5	2	2.27%
15	6B	1	1.14%
16	7C	1	1.14%
17	10A	1	1.14%
18	11A	1	1.14%
19	16F	1	1.14%
20	24F	1	1.14%
21	33B	1	1.14%
22	35B	1	1.14%
23	39	1	1.14%
24	35C	1	1.14%
25	Non-typable	2	2.27%
Total		88	100.00%

Supplementary Table 4. Serotype distribution among 175 NIPD cases.

Serial number	Serotype	No. of isolates	Constituent ratio
1	19F	28	16.00%
2	19A	22	12.57%
3	3	18	10.29%
4	6A	13	7.43%
5	23F	10	5.71%
6	23A	10	5.71%
7	15A	9	5.14%
8	14	7	4.00%
9	6B	7	4.00%
10	34	7	4.00%
11	6E	7	4.00%
12	35B	5	2.86%
13	6C	4	2.29%
14	18C	3	1.71%
15	7C	3	1.71%
16	10B	3	1.71%
17	20	2	1.14%
18	13	2	1.14%
19	16F	2	1.14%
20	33B	2	1.14%
21	9V	1	0.57%
22	15B	1	0.57%
23	9N	1	0.57%
24	39	1	0.57%
25	35C	1	0.57%
26	15BC	1	0.57%
27	31	1	0.57%
28	35F	1	0.57%
29	17A	1	0.57%
30	17F	1	0.57%
31	Non-typable	1	0.57%
Total		175	100.00%

Supplementary Table 5. Serotype distribution among 141 pediatric cases (age <18 years).

Serial number	Serotype	No. of isolates	Constituent ratio
1	19F	30	21.28%
2	19A	16	11.35%
3	6A	10	7.09%
4	6E	10	7.09%
5	14	10	7.09%
6	15A	9	6.38%
7	23A	9	6.38%
8	23F	8	5.67%
9	3	6	4.26%
10	6B	6	4.26%
11	18C	4	2.84%
12	6C	3	2.13%
13	34	3	2.13%
14	13	2	1.42%
15	10B	2	1.42%
16	16F	2	1.42%
17	33B	2	1.42%
18	35B	2	1.42%
19	5	1	0.71%
20	39	1	0.71%
21	35C	1	0.71%
22	24F	1	0.71%
23	15BC	1	0.71%
24	Non-typable	2	1.42%
Total		141	100.00%

Supplementary Table 6. Serotype distribution among 101 elderly cases (age > 50 years).

Serial number	Serotype	No. of isolates	Constituent ratio
1	3	16	15.84%
2	19F	13	12.87%
3	19A	12	11.88%
4	34	6	5.94%
5	23F	6	5.94%
6	6A	5	4.95%
7	23A	5	4.95%
8	35B	5	4.95%
9	7C	4	3.96%
10	15A	4	3.96%
11	6E	3	2.97%
12	13	2	1.98%
13	14	2	1.98%
14	20	2	1.98%
15	6B	2	1.98%
16	5	1	0.99%
17	6C	1	0.99%
18	9V	1	0.99%
19	9N	1	0.99%
20	10B	1	0.99%
21	15B	1	0.99%
22	16F	1	0.99%
23	17A	1	0.99%
24	17F	1	0.99%
25	31	1	0.99%
26	39	1	0.99%
27	35C	1	0.99%
28	35F	1	0.99%
29	Non-typable	1	0.99%
Total		101	100.00%

Supplementary Table 7. Characteristics of serotypes, STs and diagnosis of patients aged 18-50 years old.

Characteristics	No. of patients	(%)
Serotypes		
34	4	19.05
19F	4	19.05
23F	3	14.29
14	2	9.52
19A	2	9.52
1	1	4.76
3	1	4.76
10A	1	4.76
11A	1	4.76
18C	1	4.76
23A	1	4.76
STs		
271	3	14.29
320	2	9.52
615	1	4.76
673	1	4.76
870	1	4.76
876	1	4.76
4640	1	4.76
7753	1	4.76
11964	1	4.76
12944	1	4.76
16240	1	4.76
16448	1	4.76
17950	1	4.76
17962	1	4.76
18037	1	4.76
18048	1	4.76
18050	1	4.76
18051	1	4.76
Diagnosis		
Invasive pneumococcal disease (IPD)	15	71.43
Noninvasive pneumococcal disease (NIPD)	6	28.57
**Total**	21	100.00

Supplementary Table 8. Sequence type distribution among 263 *S. pneumoniae* isolates.

Serial number	STs	No. of isolates	Constituent ratio
1	271	33	12.55%
2	320	31	11.79%
3	90	11	4.18%
4	876	11	4.18%
5	11972	9	3.42%
6	902	8	3.04%
7	81	6	2.28%
8	5242	6	2.28%
9	870	5	1.90%
10	1937	5	1.90%
11	2754	5	1.90%
12	11945	5	1.90%
13	180	4	1.52%
14	338	4	1.52%
15	505	4	1.52%
16	673	4	1.52%
17	15069	4	1.52%
18	9789	3	1.14%
19	11967	3	1.14%
20	17950	3	1.14%
21	17961	3	1.14%
22	236	2	0.76%
23	242	2	0.76%
24	3173	2	0.76%
25	6011	2	0.76%
26	6227	2	0.76%
27	6327	2	0.76%
28	13646	2	0.76%
29	14094	2	0.76%
30	14702	2	0.76%
31	17951	2	0.76%
32	Others	76	28.90%
Total		263	100.00%

Others: only 1 isolate in 1 ST.

Supplementary Table 9. Clonal complexes among 263 *S. pneumoniae* isolates.

CCs/ Singletons	PMEN (SLV)	STs	Serotype	No. of isolates
CC63	Sweden^{15A}-25	ST63	15A	1
		ST1191	35B	1
CC18048		ST18048	14	1
CC4640		ST4640	34	1
		ST7753	34	1
		ST18046	34	1
CC18052		ST18052	34	1
CC271	Taiwan^{19F}-14	ST271	19F	33
		ST320	19A	29
			19F	2
		ST17965	19A	1
		ST18053	19F	1
		ST18037	19F	1
		ST18038	19F	1
		ST1937	19F	5
		ST236	19F	2
CC2754		ST2754	10B	2
			6A	1
			6C	1
			33B	1
		ST9821	6A	1
		ST17963	10B	1
		ST7752	35C	1
		ST14094	6A	2
		ST17966	6A	1
CC10392		ST10392	6C	1
CC4112		ST4112	17F	1
CC17951		ST17951	39	2
CC673		ST673	3	4
CC870		ST870	18C	5
CC18045		ST18045	33B	1
CC9789		ST9789	6A	3
CC12944		ST12944	10A	1
CC16240		ST16240	23F	1
		ST17171	24F	1
		ST17950	23F	2

			31	1
CC6542		ST6542	16F	1
		ST18042	16F	1
CC8250		ST8250	16F	1
CC18050		ST18050	11A	1
CC99		ST99	NT	1
CC615	USA[1]-29	ST615	1	1
CC17954		ST17954	NT	1
CC10085		ST10085	3	1
CC17956		ST17956	6C	1
CC471		ST471	6A	1
		ST1927	6A	1
CC1876		ST1876	6A	1
CC6011		ST6011	15A	2
		ST17960	15A	1
		ST11972	15A	9
CC4655		ST4655	3	1
		ST15069	3	4
CC17949		ST17949	3	1
CC17967		ST17967	35F	1
CC166		ST166	9V	1
CC18049		ST18049	NT	1
CC180	Netherlands[3]-31	ST180	3	4
		ST18047	3	1
CC505		ST505	3	4
		ST15272	3	1
CC11963		ST11963	35C	1
CC18040		ST18040	15BC	1
CC17959		ST17959	13	1
		ST11952	13	1
CC3397		ST3397	15B	1
CC17961		ST17961	35B	3
CC6327		ST6327	35B	2
CC7397		ST7397	6B	1
CC338	Colombia[23F]-26	ST338	23A	4
		ST5242	23A	6
CC3173		ST3173	6A	2
		ST6340	6B	1

CC17952		ST17952	33B	1
CC342		ST342	23F	1
CC13646		ST13646	23F	2
CC876		ST876	14	10
			7C	1
		ST18039	14	1
		ST17969	14	1
		ST17957	14	1
CC902		ST902	6A	2
			6B	6
CC16327		ST16327	19F	1
CC17964		ST17964	23A	1
		ST18051	23A	1
CC12656		ST12656	6C	1
CC17946		ST17946	17A	1
CC17970		ST17970	3	1
CC9114		ST9114	20	1
		ST17945	20	1
		ST17158	3	1
CC15444		ST15444	13	1
CC14151		ST14151	13	1
CC18041	Colombia^{5}-19	ST18041	5	1
CC18044		ST18044	5	1
CC17947		ST17947	9N	1
CC6227	Denmark^{14}-32	ST6227	23A	2
		ST9396	23A	1
		ST5033	23F	1
		ST230	23F	1
CC242	Taiwan^{23F}-15	ST242	23F	2
CC81	Spain^{23F}-1	ST81	23F	6
CC16448		ST16448	23F	1
CC90	Spain^{6B}-2	ST90	6E	11
		ST95	6E	1
CC13962		ST13962	6E	1
CC11945		ST11945	34	5
		ST18043	19F	1
CC11967		ST11967	7C	3
CC14702		ST14702	34	2

CC17962	ST17962	34	1
CC11964	ST11964	34	1

Supplementary Table 10. Antibiotic resistance of 263 *S. pneumoniae* isolates.

Antibiotic		S%	I%	R%	MIC_{50} (µg/mL)	MIC_{90} (µg/mL)	MIC range (µg/mL)
PEN	(meningitis)	17.65	0.00	82.35	1.00	2.80	≤0.06-≥8.00
	(nonmeningitis)	93.90	4.88	1.22	0.50	2.00	≤0.06-≥8.00
AMX		80.23	9.89	9.89	≤2.00	4.00	≤2.00-≥8.00
CTX		88.97	0.00	11.03	≤0.50	≥4.00	≤0.05-≥4.00
MEN		57.41	33.46	9.13	≤0.25	0.50	≤0.25-≥1.00
VAN		100.00	0.00	0.00	≤1.00	≤1.00	<0.25-≥2.00
ERY		3.04	0.00	96.96	≥1.00	≥1.00	≤0.25-≥1.00
OFX		95.82	3.04	1.14	2.00	2.00	≤1.00-≥8.00
LVX		98.48	0.38	1.14	≤2.00	≤2.00	≤2.00-≥8.00
MFX		99.24	0.38	0.38	≤0.50	≤0.50	≤0.50-≥4.00
TET		19.01	1.14	79.85	16.00	16.00	≤1.00-32.00
SXT		28.14	12.93	58.94	≥4/76	≥4/76	≤0.5/9.5-≥4/76
LNZ		100.00	0.00	0.00			

MIC: Minimum inhibitory concentration, S: Sensitive, I: Intermediary, R: Resistant.

Supplementary Table 11. Relationship between antibiotic resistance and serotypes.

Antibiotic		R%														
		**19F** (n=47)	**19A** (n=30)	**3** (n=23)	**23F** (n=17)	**6A** (n=15)	**23A** (n=15)	**14** (n=14)	**34** (n=13)	**15A** (n=13)	**6E** (n=13)	**6B** (n=8)	**35B** (n=6)	**6C** (n=4)	**13** (n=4)	**7C** (n=4)
PEN	(meningitis)	100.00	100.00	/	100.00	/	/	100.00	0.00	100.00	100.00	/	/	/	/	/
	(nonmeningitis)	0.00	3.45	0.00	0.00	0.00	0.00	0.00	8.33	0.00	0.00	0.00	0.00	0.00	0.00	0.00
AMX		34.04	20.00	0.00	5.88	0.00	6.67	0.00	7.69	0.00	0.00	0.00	0.00	0.00	0.00	0.00
CTX		38.30	13.33	0.00	5.88	0.00	6.67	7.14	15.38	7.69	0.00	0.00	0.00	0.00	0.00	0.00
MEM		19.15	23.33	0.00	11.76	0.00	0.00	14.29	7.69	15.38	0.00	0.00	0.00	0.00	0.00	0.00
VAN		0.00	0.00	0.00	0.00	0.00	0.00	0.00	0.00	0.00	0.00	0.00	0.00	0.00	0.00	0.00
ERY		95.74	96.67	95.65	100.00	100.00	100.00	100.00	100.00	100.00	100.00	100.00	100.00	100.00	100.00	100.00
OFX		0.00	0.00	0.00	0.00	0.00	0.00	0.00	0.00	7.69	0.00	0.00	0.00	0.00	0.00	25.00
LVX		0.00	0.00	0.00	0.00	0.00	0.00	0.00	0.00	7.69	0.00	0.00	0.00	0.00	0.00	25.00
MFX		0.00	0.00	0.00	0.00	0.00	0.00	0.00	0.00	7.69	0.00	0.00	0.00	0.00	0.00	0.00
TET		80.85	83.33	65.22	76.47	73.33	86.67	92.86	69.23	84.62	76.92	100.00	100.00	100.00	75.00	75.00
SXT		85.11	96.67	17.39	76.47	60.00	40.00	21.43	61.54	7.69	92.31	25.00	16.67	50.00	50.00	75.00
LNZ		0.00	0.00	0.00	0.00	0.00	0.00	0.00	0.00	0.00	0.00	0.00	0.00	0.00	0.00	0.00

Supplementary Table 12. Results comparison of resistance percentage between the previous report (Yan et al., 2021) and the current report.

Antibiotic	Previous report	Current report	*P*
PEN	5.47% (7/128)	6.84% (18/263)	0.602
CTX	10.94% (14/128)	11.03% (29/263)	0.979
AMX	3.13% (4/128)	8.75% (23/263)	0.078
ERY	2.34% (3/128)	6.46% (17/263)	1.000
LVX	9.38% (12/128)	5.70% (15/263)	0.223
TET	3.13% (4/128)	5.70% (15/263)	0.829
SXT	7.81% (10/128)	5.32% (14/263)	0.499

Supplementary Table 13. Relationship between antibiotic resistance and top prevalent STs.

Antibiotic		R%						*P*
		**ST271** (n=33)	**ST320** (n=31)	**ST90** (n=11)	**ST876** (n=11)	**ST11972** (n=9)	**ST902** (n=8)	
PEN	(meningitis)	100.00	100.00	100.00	100.00	100.00	/	NA
	(nonmeningitis)	3.03	3.23	0.00	9.09	0.00	0.00	0.003
AMX		45.45	22.58	0.00	0.00	0.00	0.00	0.000
CTX		45.45	12.90	0.00	9.09	11.11	0.00	0.000
MEM		24.24	22.58	0.00	0.00	22.22	0.00	0.000
VAN		0.00	0.00	0.00	0.00	0.00	0.00	NA
ERY		100.00	96.77	100.00	100.00	100.00	100.00	0.859
OFX		0.00	0.00	0.00	0.00	11.11	0.00	0.874
LVX		0.00	0.00	0.00	0.00	11.11	0.00	1.000
MFX		0.00	0.00	0.00	0.00	11.11	0.00	0.376
TET		81.82	83.87	90.91	90.91	88.89	100.00	0.986
SXT		93.94	96.77	90.91	18.18	11.11	0.00	0.000
LNZ		0.00	0.00	0.00	0.00	0.00	0.00	NA
